# Haloperidol Metabolite II Valproate Ester (*S*)-(−)-MRJF22: Preliminary Studies as a Potential Multifunctional Agent Against Uveal Melanoma

**DOI:** 10.1021/acs.jmedchem.1c00995

**Published:** 2021-09-03

**Authors:** Carla Barbaraci, Giovanni Giurdanella, Claudia Giovanna Leotta, Anna Longo, Emanuele Amata, Maria Dichiara, Lorella Pasquinucci, Rita Turnaturi, Orazio Prezzavento, Ivana Cacciatore, Elisa Zuccarello, Gabriella Lupo, Giovanni Mario Pitari, Carmelina Daniela Anfuso, Agostino Marrazzo

**Affiliations:** † Department of Drug and Health Sciences, University of Catania, Viale A. Doria 6, 95125 Catania, Italy; ‡ Department of Biomedical and Biotechnological Sciences, School of Medicine, University of Catania, Via S. Sofia 97, 95123 Catania, Italy; § Vera Salus Ricerca S.r.l., Via Sigmund Freud 62/B, 96100 Siracusa, Italy; ∥ Department of Pharmacy, “G. D’Annunzio” University of Chieti-Pescara, Via dei Vestini 31, 66100 Chieti Scalo, Italy; ⊥ Taub Institute for Research on Alzheimer’s Disease and the Aging Brain, Columbia University, New York, New York 10032, United States

## Abstract

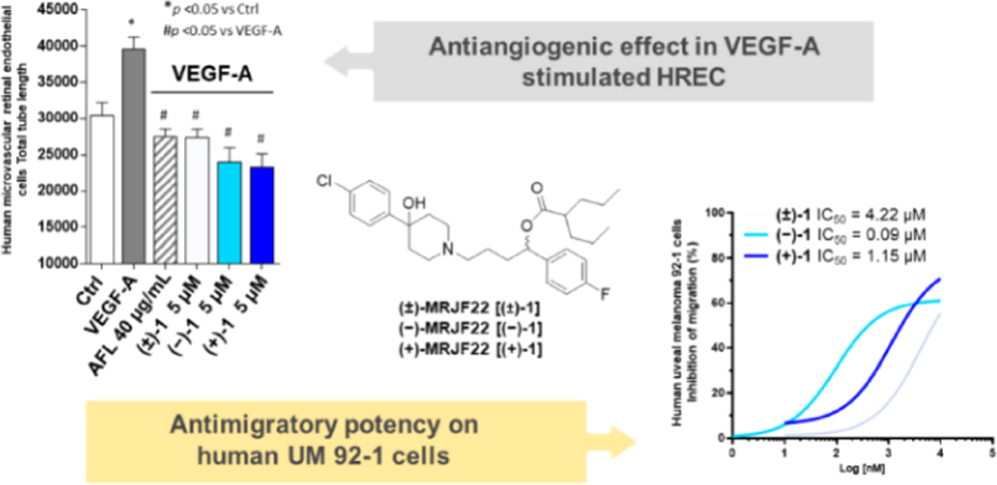

Increased angiogenesis and vascular endothelial growth factor (VEGF) levels contribute to higher metastasis and mortality in uveal melanoma (UM), an aggressive malignancy of the eye in adults. **(±)-MRJF22**, a prodrug of the sigma (σ) ligand haloperidol metabolite II conjugated with the histone deacetylase (HDAC) inhibitor valproic acid, has previously demonstrated a promising antiangiogenic activity. Herein, the asymmetric synthesis of **(R)-(+)-MRJF22** and **(S)-(−)-MRJF22** was performed to investigate their contribution to **(±)-MRJF22** antiangiogenic effects in human retinal endothelial cells (HREC) and to assess their therapeutic potential in primary human uveal melanoma (UM) 92-1 cell line. While both enantiomers displayed almost identical capabilities to reduce cell viability than the racemic mixture, **(S)-(−)-MRJF22** exhibited the highest antimigratory effects in endothelial and tumor cells. Given the fundamental contribution of cell motility to cancer progression, **(S)-(−)-MRJF22** may represent a promising candidate for novel antimetastatic therapy in patients with UM.

## Introduction

Uveal melanoma (UM) is a rare and aggressive intraocular tumor, which arises from melanocytes such as cutaneous melanoma, but presents unique biology and genetic traits.^1^ Approximately 90% of all UMs involve the choroid, while the rest involve the ciliary body (6%) or iris (4%).^2^ Despite early diagnosis and treatment with conventional chemotherapy and surgery, nearly 50% of all UM patients develop hepatic metastases, which are usually fatal within 1 year from diagnosis.^3−5^

Lacking an intraocular lymphatic system, UM tends to spread via a hematogenous route, and the presence of microvascular loops and networks is clinically related to UM progression and a worse prognosis.^6^ Recent studies have shown that different UM cell lines produce a copious amount of vascular endothelial growth factor (VEGF), the primary activator of tumor angiogenesis in mammals.^9^ Treatment of inoperable UM patients with the VEGF-trap aflibercept (AFL), a popular drug in ophthalmology, resulted in 50% progression-free survival at 4 months.^7−9^ Intravitreal injection of bevacizumab, an antiangiogenic monoclonal antibody targeting all isoforms of vascular endothelial growth factor A (VEGF-A), is currently under evaluation through a phase II trial for the treatment of UM metastatic disease.^10−12^

Sigma (σ) receptors are involved in different biological functions, including cell proliferation and survival, and are overexpressed in several tumor cell lines.^13^ This unique class of receptors consists of two subtypes, sigma-1 (σ_1_) and sigma-2 (σ_2_). The σ_1_ receptor is a chaperon protein at the mitochondria-associated membrane (MAM) involved in apoptosis, and its in vivo silencing modulates endothelial cell proliferation and inhibits angiogenesis.^14^ The presence of the σ_1_ receptor in cancer cells increases VEGF secretion and stimulates motility, in part through the regulation of the human voltage-dependent K^+^ channel (hERG) membrane expression.^15^ Recently identified as the ER-resident transmembrane protein 97 (TMEM97), the σ_2_ receptor is poorly understood.^16^ Recognized as a biomarker of cell proliferation, the mechanism by which the σ_2_ receptor promotes apoptosis and autophagy remains unclear.^17,18^ Data suggest that σ receptors are able to induce apoptosis and autophagy in UM, whereas they mediate opposite biological effects on cell proliferation.^19,20^ Notably, compounds endowed with a σ_1_ receptor antagonists/σ_2_ receptor agonist functional profile such as haloperidol (HP) and haloperidol metabolite II (HP-mII) have been shown to reduce human UM cell proliferation. Different from HP-mII, which displays a preferential activity for σ receptors compared to other receptor systems, antiproliferation by HP on UM 92-1 cells may be due to additional nonspecific effects.^20,21^

Histone deacetylases (HDACs) are enzymes involved in specific epigenetic changes associated with cancer and other diseases.^22^ Inhibition of HDACs induces hyperacetylation of histones, which affects gene expression.^23^ HDAC inhibitors (HDACis) induce the inhibition of angiogenesis through various mechanisms, such as activation of cell-cycle arrest or induction of apoptosis and autophagy.^22,24,25^ More than 20 HDACis have entered clinical studies, with vorinostat and romidepsin approved for the treatment of cutaneous T-cell lymphoma.^26,27^ HDACi may play a role in the adjuvant therapy of patients with UM by inducing differentiation and prolonged dormancy of micrometastases.^28^ In this context, HDACi valproic acid (VPA) induces G1 cell-cycle arrest of UM cells and reduces UM progression in vivo and has recently undergone a phase II clinical trial for high-risk UM patients.^29−31^

**Figure 1:**
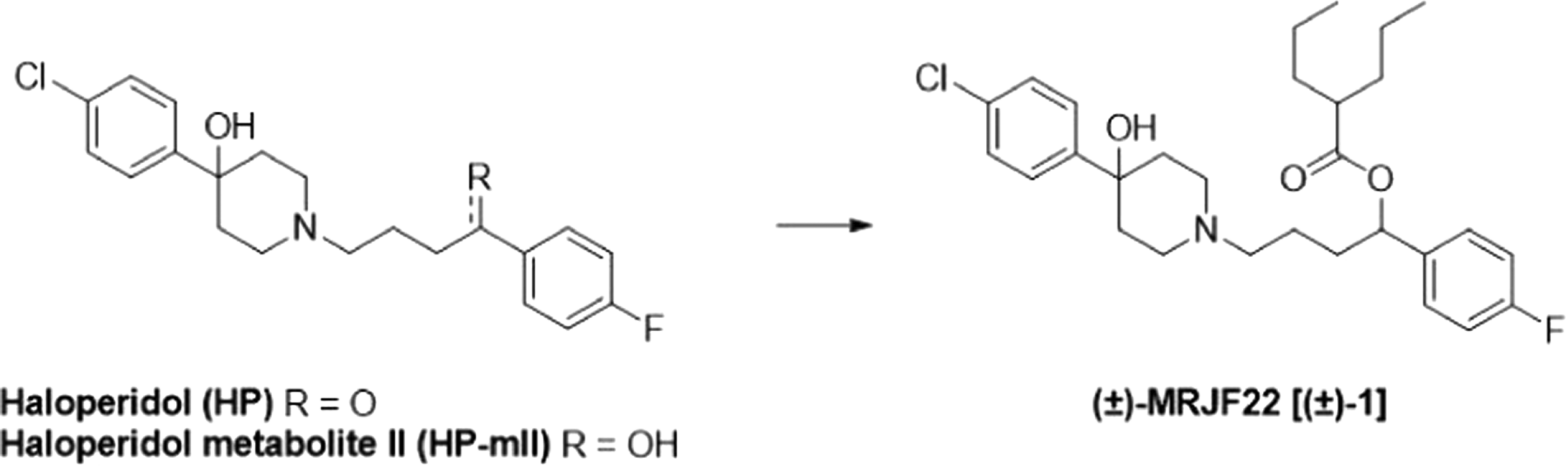
Chemical structure of HP, HP-mII, and **(±)-MRJF22**.

To improve antiangiogenic and anticancer capabilities, dual ligands targeting σ receptors and HDAC were previously developed employing a prodrug approach.^32,33^**(±)-MRJF22** [**(±)-1**], a prodrug of (±)-HP-mII with VPA (Figure 1), significantly reduced cell migration and proliferation (20 and 120 times more than (±)-HP-mII and VPA, respectively) in VEGF-A-stimulated human retinal endothelial cells (HRECs).^34^

Angiogenesis within UM progression is the result of a complex interplay between endothelial and tumor cells, in which VEGF-A may play a significant role.^35,36^ The involvement of σ receptors and HDAC in antiangiogenic and antiproliferative activities makes the prodrug **(±)-1** a potential pharmacological tool exploitable for the treatment of UM metastatic disease. To gain more insights and define contribution to antiangiogenic effects by **(±)-1**, the asymmetric synthesis of **(R)-(+)-MRJF22** [**(+)-1**] and **(S)-(−)-MRJF22** [**(−)-1**] and their evaluation on VEGF-A-stimulated HRECs is described here. Moreover, present studies investigate the effects of **(±)-1** and its enantiomers on human UM 92-1 cell proliferation and migration, which together with angiogenesis represents the principal determinants of metastasis development in UM.

## Results and Discussion

### Chemistry

**Scheme 1:**
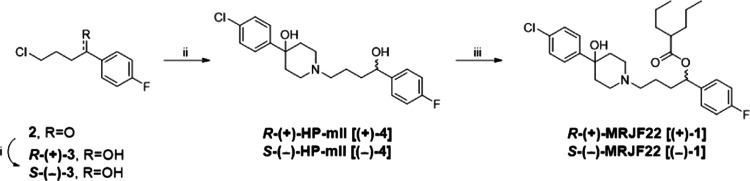
Enantioselective Synthesis for Compounds **(+)-1** and **(−)-1** Reagents and conditions: (i) (+)- or (−)-DIP-Cl, tetrahydrofuran (THF), −25 °C, 16 h; DEA, Et_2_O, room temperature (rt), on; (ii) 4-(4-chlorophenyl)hydroxypiperidine, KHCO_3_, anhydrous dimethylformamide (DMF), 80 °C, 24 h; (iii) 2-propylpentanoyl chloride, THF, 0 °C to rt, 3 h.

Synthesis of **(+)-1** and **(−)-1** was achieved through enantioselective reduction, as reported in the literature.^33,37^ According to the steps in Scheme 1, the commercially available 4-chloro-1-(4-fluorophenyl)butan-1-one (**2**) was treated with the reductive agents (+) or (**−**)-diisopinocampheylchloroborane (DIP-Cl), allowing a highly stereoselective reaction. Afterward, diethanolamine (DEA) was added, and the intermediates **(+)-3** and **(−)-3** were used for the synthesis of compounds (*R*)-(+)-HP-mII [**(+)-4**] and (*S*)-(−)-HP-mII [**(−)-4**] by nucleophilic substitution on the nitrogen of the amine 4-(4-chlorophenyl)hydroxypiperidine. The 2-propylpentanoyl chloride was allowed to react with compounds **(+)-4** and **(−)-4**, giving compounds **(+)-1** and **(−)-1**.^34^

The optical rotation for both enantiomers was determined in CHCl_3_ solution to afford **(+)-1** [α]_D_^20^ = +24.8° (*c*1.0, CHCl_3_) and **(−)-1** [α]_D_^20^ = −26.0° (*c*1.0, CHCl_3_).

Enantiomeric excess (ee) was calculated to measure the purity of the synthesized compound by high-performance liquid chromatography (HPLC) analyses using a Chiralcel OJ[-RH] column. Both enantiomers were obtained enantiomerically pure, showing an ee equal to 92 and 95.4% for **(+)-1** and **(−)-1**, respectively (Figure S1, Supporting Information).

### Chemical and Enzymatic Stability

The chemical stability of **(±)-1** was evaluated in buffer solutions at pH 1.3 and 7.4 (Table 1). The results showed that the racemate was stable for about 4 days in both environments.

**Table 1:** Chemical and Enzymatic Stabilities of **(±)-1** and Its Two Enantiomers

		**(±)-1**		**(+)-1**		**(−)-1**	
stability		*t*_1/2_ (h)	*K*_obs_ (h^–1^)	*t*_1/2_ (h)	*K*_obs_ (h^–1^)	*t*_1/2_ (h)	*K*_obs_ (h^–1^)
chemical^a^	pH 1.3	100.1 ± 3.1	0.007 ± 0.001				
	pH 7.4	96.4 ± 1.6	0.007 ± 0.001				
enzymatic^a^	human plasma	60.5 ± 0.6	0.011 ± 0.006	58.2 ± 1.2	0.012 ± 0.001	70.3 ± 1.5	0.01 ± 0.002
	rat plasma	0.234 ± 0.003	2.96 ± 0.01	0.434 ± 0.002	1.60 ± 0.005	0.322 ± 0.01	2.15 ± 0.03

a Values are means ± standard deviation (SD) of three experiments.

Enzymatic stability studies in rat and human plasma were performed for both the racemate and its enantiomers (Table 1). As we expected, the compounds were rapidly hydrolyzed by carboxylesterases present in rat plasma (*t*_1/2_, 0.2–0.4 h) compared to human plasma (*t*_1/2_, >58 h) due to the huge disparity between the esterase content in both plasma.^38^ Considering the high stability of **(±)-1** and its enantiomers in simulated physiological fluids and human plasma, these compounds could reach the target site without undergoing esterase metabolism.

### Sigma Receptor Binding Assay

To examine the effects of asymmetric synthesis on σ receptor occupancy, σ_1_ and σ_2_ receptor affinities for **(+)-1** and **(−)-1** were measured and compared to precursors **(+)-4** and **(−)-4** and their respective racemic mixtures (Table 2).

**Table 2:** σ_1_ and σ_2_ Binding Assays for Compound **(±)-1**, Its Enantiomers, and Precursor Compounds

	*K*_i_ ± SD (nM)^a^	
cmpd	σ_1_	σ_2_
**(±)-1**^b^	13 ± 0.6	124 ± 15
**(+)-1**	64 ± 8.2	74 ± 8.9
**(−)-1**	16 ± 1.7	56 ± 6.4
(±)-HP-mII^b^	2.9 ± 1.1	2.4 ± 0.7
**(+)-4**^b^	2.0 ± 0.6	32 ± 2.8
**(−)-4**^b^	3.0 ± 1.1	9.8 ± 1.8
HP^b^	2.7 ± 0.7	17 ± 0.7

a Each value is the mean ± SD of at least two experiments performed in duplicate.

b Data taken from ref (34).

The esterification of the secondary hydroxy group of **(+)-4** and **(−)-4** with VPA decreased the binding affinity of σ receptors compared to (±)-HP-mII, confirming previous observations with **(±)-1**.^34^ Distinctive differences emerged for the enantiomer binding profiles at σ receptors. The σ_1_ binding affinity of the (−)-enantiomer (*K*_i_ = 16 nM) was similar to that of **(±)-1** (*K*_i_ = 13 nM) but higher than that of the (+)-stereoisomer (*K*_i_ = 64 nM). Both enantiomers **1** exhibited higher affinities for σ_2_ receptors than the racemic mixture **(±)-1** with a *K*_i_ value of 56 nM for **(−)-1** and 74 nM for **(+)-1**. In contrast, their precursors **(+)-4** and **(−)-4** exhibited lower affinities for σ_2_ receptors than (±)-HP-mII, probably due to positive allosteric modulations of the two enantiomers for σ receptors.^39^ The superior binding affinity of the **(−)-1** enantiomer on the σ_2_ receptor probably reflects favorable molecular interactions by the precursor **(−)-4** with respect to the **(+)**-enantiomer or HP.

### Antiangiogenic Activity on HREC

To dissect enantiomer contributions to antiangiogenic effects by **(±)-1**, compounds **(+)-1** and **(−)-1** were evaluated on HREC, an endothelial cell model of angiogenesis.^40^ First, HREC viability was assessed by the 3-[4,5–dimethylthiazol-2-yl]-2,5-diphenyltetrazolium bromide (MTT) assay to verify the tolerability of the enantiomers, and different concentrations (1.0, 2.5, 5.0, 10.0, and 20.0 μM) of the compounds were tested for 24, 48, and 72 h. The IC_50_ values of **(+)-1** and **(−)-1** were calculated and compared to **(±)-1** and its precursors (Table 3).

**Table 3:** IC_50_ Obtained by the MTT Viability Test on HREC at Different Time Points for Compounds **(+)-1** and **(−)-1** and Their Precursors

cmpd	time (h)	IC_50_ (μM)^a^	pIC_50_ ± SE^b^
**(±)-1**^c^	24	10.5	4.98 ± 0.02
	48	11.1	4.95 ± 0.03
	72	10.1	4.99 ± 0.04
**(+)-1**	24	9.8	5.01 ± 0.03
	48	6.4	5.19 ± 0.01
	72	4.4	5.35 ± 0.01
**(−)-1**	24	10.1	4.97 ± 0.07
	48	9.0	5.04 ± 0.01
	72	6.8	5.16 ± 0.42
VPA^c^	24	1217	2.91 ± 0.01
	48	1449	2.84 ± 0.03
	72	1393	2.86 ± 0.01
(±)-HP-mII^c^	24	>200^d^	
	48	128	3.89 ± 0.04
	72	69	4.16 ± 0.03
HP^c^	24	3.7	5.43 ± 0.04
	48	2.9	5.54 ± 0.08
	72	2.6	5.59 ± 0.07

a IC_50_ values have been calculated with GraphPad Prism 5 for Windows using a nonlinear fit transform sigmoidal dose–response (variable slope). IC_50_ values are averaged from multiple determinations (*n* = 3, each of them).

b pIC_50_ is defined as the −log(IC_50_).

c Data taken from ref (34).

d Cell viability reduction lower than 50% at 200 μM.

At 24 h, the enantiomers showed similar extent and efficacy in reducing HREC cell viability compared to the racemic mixture **(±)-1** but, contrary to this, reduced the cell viability in a time-dependent manner (Figure S2). Indeed, the IC_50_ ranged from 9.8 to 4.4 μM for **(+)-1** and from 10.1 to 6.8 μM for **(−)-1**, not significantly different from those of **(±)-1** (from 10.5 to 10.1 μM). Moreover, confirming previous results with **(±)-1**,^34^ both enantiomers exhibited higher antiproliferative potencies in HREC with respect to precursors VPA and (±)-HP-mII (Table 3). In contrast, HP remains the most potent compound in reducing HREC viability, compared to **(±)-1** and its enantiomers (Table 3).^34^ The results suggest that the enantiomers exert a toxicity comparable to that of the racemic mixture on HREC and, in any case, more tolerated by the cells than their precursor HP.

**Figure 2:**
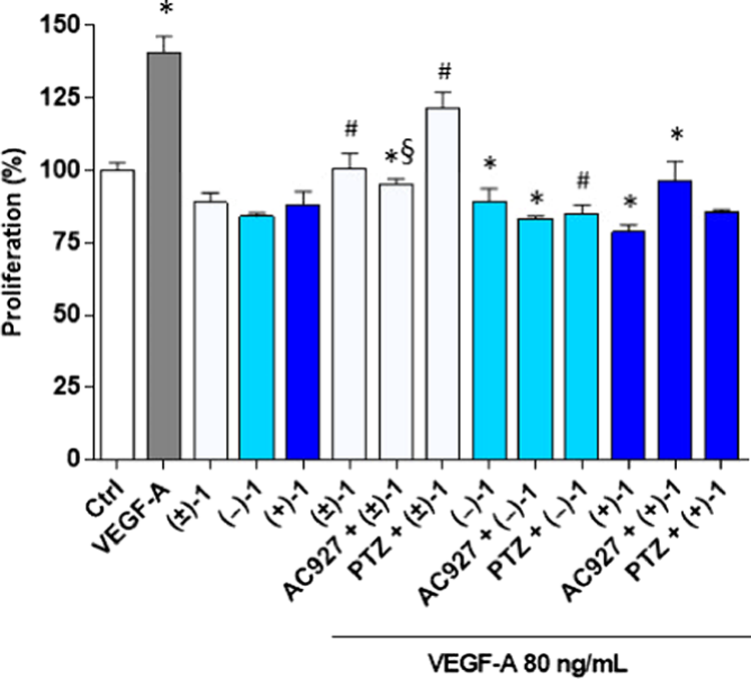
Antiproliferative effects of **(±)-1**, **(+)-1,** and **(−)-1** in HREC stimulated with VEGF-A, assessed by the crystal violet assay. HREC were treated with 5 μM **(±)-1**, **(+)-1,** and **(−)-1** in the presence or absence of 80 ng/mL VEGF-A for 24 h. Effects of the σ_2_ receptor antagonist AC927 (2 μM) and the selective σ_1_ receptor agonist (+)-pentazocine (PTZ) (2 μM) in HREC cotreated with 5 μM **(±)-1** or **(+)-1** and **(−)-1** 80 ng/mL of VEGF-A for 24 h. Ctrl, vehicle control (dimethyl sulfoxide, DMSO). Data are expressed as a percentage of proliferation with respect to vehicle control. *p < 0.05 vs Ctrl; ^#^*p* < 0.05 vs VEGF-A; ^§^*p* < 0.05 vs the same conditions without agonist or antagonist.

To investigate the ability of **(±)-1**, **(+)-1**, and **(−)-1** to counteract the VEGF-A proangiogenic effect, crystal violet assays were carried out to evaluate cell proliferation in HREC stimulated with 80 ng/mL VEGF-A (Figure 2). As expected, VEGF-A exerted a proangiogenic effect by increasing cell proliferation by about 45% (at 24 h) with respect to the untreated control (Figure 2). Under basal conditions, treatments with 5 μM **(±)-1**, **(+)-1**, and **(−)-1** did not induce significant changes in HREC proliferation rates after 24 h (Figure 2). In contrast, equimolar concentrations (5 μM) of **(±)-1**, **(+)-1,** and **(−)-1** completely prevented VEGF-A-mediated HREC proliferation (Figure 2). These data are in agreement with previous reports obtained with **(±)-1**, which demonstrated that this prodrug exhibits peculiar antiangiogenetic effects comparable to bevacizumab that are not shared with the precursors VPA and (±)-HP-mII.^34^

To evaluate the role of σ receptors in prodrug-mediated inhibition of VEGF-A-stimulated HREC proliferation, **(±)-1** and its enantiomers were examined in combination with the σ_1_ receptor agonist (+)-pentazocine [PTZ, 2 μM] and the σ_2_ receptor antagonist 1-phenethylpiperidine (AC927, 2 μM).

Confirming previous results,^34^ PTZ partially counteracted the anti-VEGF-A activity of the **(±)-1** racemic mixture (Figure 2), suggesting that **(±)-1** may function, at least in part, as a σ_1_ antagonist in the inhibition of VEGF-A-stimulated HREC proliferation. Surprisingly, coincubations with PTZ or AC927 did not alter the anti-VEGF-A effects on proliferation by **(+)-1** and **(−)-1** (Figure 2), ruling out a relevant role of σ receptor in antiangiogenetic effects by the prodrugs with respect to VEGF-A-mediated HREC proliferation. Presumably, a minimal contribution of σ_1_ receptors to racemic mixture effects (Figure 2) could reflect differences in overall affinities toward σ_1_ and σ_2_ receptors compared to the single enantiomer components.

**Figure 3:**
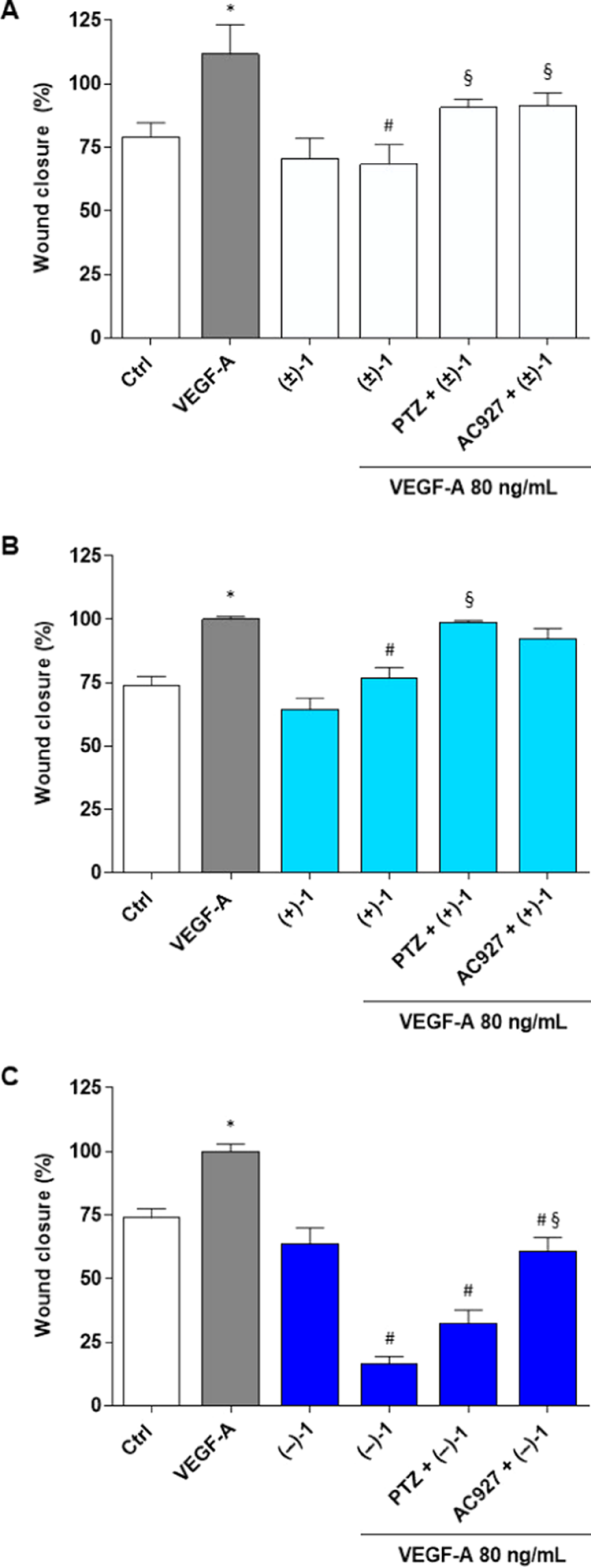
Evaluation of cell motility by wound healing assays in HREC treated with 80 ng/mL VEGF-A in the presence or absence of 5 μM **(±)-1** (A), **(+)-1** (B), and **(−)-1** (C) at 48 h. Selective σ_1_ receptor agonist PTZ (2 μM) or σ_2_ receptor antagonist AC927 (2 μM) was tested in cotreated HREC with 80 ng/mL of VEGF-A and **(±)-1**, **(+)-1**, or **(−)-1**. Wound closure percentage was quantified by ImageJ software. Ctrl, vehicle control (DMSO). Values are expressed as a mean ± standard error of the mean (SEM) of three independent experiments, each involving three different wells per condition. Statistical analysis was performed using one-way analysis of variance (ANOVA), followed by Tukey’s test. *p < 0.05 vs Ctrl; ^#^*p* < 0.05 vs VEGF-A; ^§^*p* < 0.05 vs the same conditions without agonist or antagonist.

Unregulated stimulation of endothelial cell motility by VEGF-A underlies pathological angiogenesis.^41^ Therefore, the effects of **(±)-1**, **(+)-1**, and **(−)-1** on HREC motility stimulated by VEGF-A (80 ng/mL) were evaluated with the wound healing assays (Figures 3 and S3–S5). VEGF-A promoted HREC migration compared to untreated controls, inducing complete wound closures at 24 h (Figures S3–S5). In contrast, HREC monolayers did not close their wounds over 48 h incubation in the presence of 5 μM **(±)-1** or its enantiomers (Figure 3A–C). Of note, while HREC monolayers did not significantly alter wound changes in unstimulated cells, the three prodrugs completely abrogated VEGF-A-mediated induction of HREC migration (Figure 3A–C). Cotreatments with both AC927 and PTZ reduced **(±)-1** and **(+)-1** abilities to inhibit VEGF-A-mediated wound healing (Figure 3A,B). However, while PTZ or AC927 exhibited similar actions with **(±)-1** and significantly, but partially, opposed the inhibition of VEGF-A-mediated cell motility by the racemic mixture (Figure 3A), **(+)-1** effects were significantly and completely blocked only by (+)-PTZ coincubation (Figure 3B). Distinctively, the inhibition of VEGF-A-mediated HREC motility by **(−)-1** was prevented, in part, only by cotreatment with the σ_2_ receptor antagonist AC927 (Figure 3C). These data suggest a plausible involvement of both σ_1_ and σ_2_ receptors in the regulation of VEGF-A-mediated HREC motility by the racemic mixture, reflecting the contribution of σ_1_ and σ_2_ receptor regulation by enantiomer **(+)-1** and enantiomer **(−)-1**, respectively. Moreover, compared to the other prodrugs, enantiomer **(−)-1** produced the highest reduction of HREC motility stimulated by VEGF-A (Figure 3A–C), below the basal levels of the untreated control (Figure 3C). Since **(−)-1** has the greatest σ_2_ binding affinity of the dual-ligand prodrugs (Table 2), these results suggest that σ receptor-mediated regulation of HREC migration by VEGF-A is principally sensitive to σ_2_ agonist activity. The representative images of HREC treated with VEGF-A and three prodrug derivatives after the scratch wound are shown in the Supporting Information (Figures S2–S4A).

**Figure 4:**
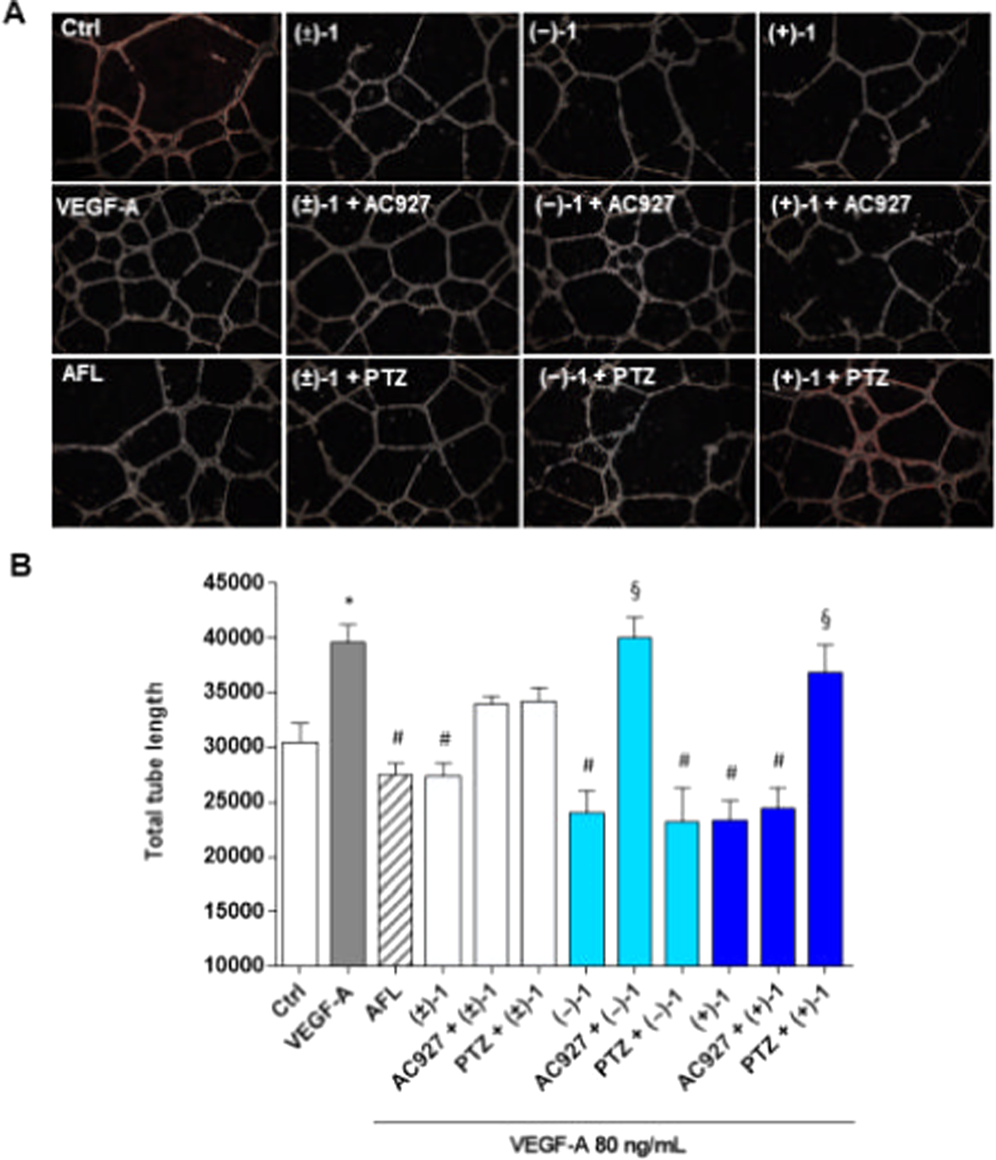
Effects **(±)-1**, **(+)-1**, and **(−)-1** on tubelike structures formed by HREC stimulated with VEGF-A. Representative optical phase-contrast micrographs of tubelike structures (40× magnification) observed in the tube formation assays (Matrigel) at 24 h (A). Quantification of tube length was carried out using the Angiogenesis Analyzer tool for ImageJ software. HREC were treated with 80 ng/mL VEGF-A in the presence or absence of 5 μM **(±)-1**, **(+)-1,** and **(−)-1** or further supplemented with selective σ_1_ receptor agonist PTZ (2 μM) and σ_2_ receptor antagonist AC927 (2 μM). We included HREC treated with 40 μg/mL of AFL (B). Values are expressed as mean ± SEM of three independent experiments, each conducted in triplicate. Statistical analysis was performed using one-way ANOVA, followed by Tukey’s test. *p < 0.05 vs Ctrl; ^#^*p* < 0.05 vs VEGF-A; ^§^*p* < 0.05 vs the same condition without agonist or antagonist.

To further explore effects on angiogenesis, tube formation assays with HREC were evaluated. Following seeding onto basement membranelike Matrigel and stimulation for 24 h with VEGF-A (80 ng/mL), HREC organized into networks of tubular structures mimicking capillary formation in neoangiogenesis (Figure 4A). As expected, VEGF-A significantly increased total tube length by HREC, an effect prevented by cotreatment with VEGF-trap AFL (Figure 4B). Treatments with **(±)-1**, **(+)-1,** or **(−)-1** also blocked VEGF-A-stimulated tube formation in HREC, with effects similar to those of AFL (Figure 4B). In close agreement with findings in HREC motility (Figure 3), while **(±)-1** actions on VEGF-A-stimulated tube formation were partially and equally blocked by both σ_1_ receptor agonist PTZ and σ_2_ receptor antagonist AC927, **(+)-1** and **(−)-1** effects were prevented selectively by AC927 and PTZ, respectively (Figure 4B). Again, these results suggest the simultaneous involvement of σ_1_ and σ_2_ receptors in the regulation of VEGF-A-induced HREC tube formation by the racemic mixture, reflecting the selective contribution of the σ_1_ receptor antagonism by enantiomer **(+)-1** and the σ_2_ receptor agonism by enantiomer **(−)-1**.

Overall, **(±)-1** and its enantiomers exhibit significant but distinct antiangiogenic effects as assessed with the HREC model in vitro. On the one hand, all three dual-ligand prodrugs induce HREC cytotoxicity and inhibit VEGF-A-mediated HREC proliferation with greater potency than precursor compounds, mostly by inducing molecular mechanisms independent of σ receptor signaling (Table 3 and Figure 2). In contrast, **(±)-1**, **(+)-1**, and **(−)-1** inhibit VEGF-A-stimulated HREC motility and tubelike structure formation stimulated by VEGF-A through the regulation of σ receptor pathways (Figures 3 and 4). In particular, the actions of enantiomers **(+)-1** and **(−)-1** appear to be selectively mediated by σ_1_ and σ_2_ receptor signaling, respectively (Figures 3 and 4). Accordingly, the enantiomer mixture **(±)-1** affects VEGF-A-mediated HREC migration and tube formation by regulating simultaneously both σ_1_ and σ_2_ receptors (Figures 3 and 4). Our data highlighted a similar aptitude of **(±)-1** and **(+)-1** in counteracting the VEGF-A proangiogenic effect through a σ_1_ receptor antagonist profile, as indicated by the PTZ capability to mitigate or completely block their effects. However, the **(−)-1** enantiomer displays the strongest antiangiogenic activity, probably as a consequence of the superior binding activity as a σ_2_ receptor agonist compared to the other dual-ligand prodrugs (Table 2). The superior inhibitory ability of **(−)-1** against VEGF-A-stimulated HREC motility is of great significance, given the established role of cell migration and invasion in metastatic UM progression.^42^

### Antiproliferative Activity on Human Uveal Melanoma 92-1 Cells

**Figure 5:**
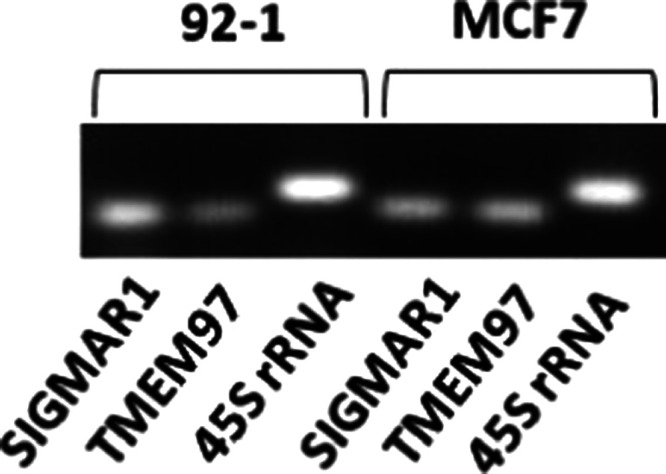
RT-PCR for the σ_1_ (SIGMAR1) and σ_2_ (TMEM97) receptor expression in UM 92-1 cells and MCF-7 human breast cancer cells. 45S Ribosomal pre-RNA was used as the positive control.

To evaluate signaling directly on UM cancer cells, the pharmacological effects of **(±)-1**, **(+)-1,** and **(−)-1** were examined in human UM 92-1 cells. First, the presence of prodrug target molecules in the 92-1 tumor cell line was checked. HDACs are ubiquitous proteins expressed in all cell types, including UM 92-1 cells.^43^ Gene expressions of σ_1_ and σ_2_ receptors, in turn, were confirmed by reverse transcription polymerase chain reaction (RT-PCR). Similar to MCF-7 human breast cancer cells (used as positive controls), UM 92-1 cells possess transcripts for both the SIGMAR1 gene (σ_1_ receptor) and the TMEM97 gene (σ_2_ receptor), a novel finding in the field (Figure 5).

**Figure 6:**
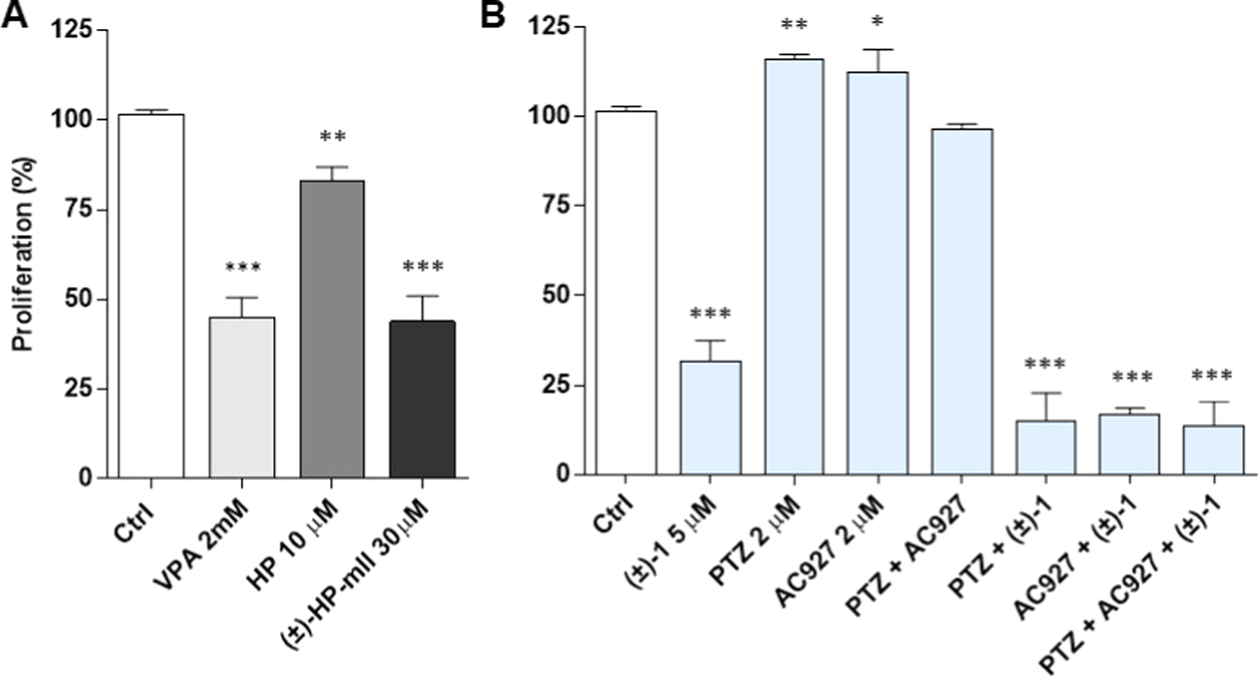
Effects of VPA (2 mM), HP (10 μM), and (±)-HP-mII (30 μM) on 92-1 cell proliferation (A). Antiproliferative effects of (±)-1 (5 μM) in combination with the selective σ_1_ receptor agonist PTZ (2 μM) and σ_2_ receptor antagonist AC927 (2 μM). Ctrl, vehicle control DMSO (B). Data in A and B represent the percentage of proliferation with respect to the vehicle control. Values are expressed as mean ± SEM of four independent experiments, each conducted in triplicate. Statistical analysis was performed using one-way ANOVA, followed by Tukey’s test. *p < 0.05; **p < 0.01; ***p < 0.001 vs vehicle control.

Then, actions on cell proliferation were examined by crystal violet staining (Figure 6). VPA, (±)-HP-mII, and HP, as the precursor and component compounds of **(±)-1**, were employed at the predicted IC_50_ values and significantly inhibited 92-1 cell proliferation compared to the vehicle control (Figure 6A).^32,44^ However, by comparing precursor data with estimated IC_50_ values of prodrugs [5.46 μM for **(±)-1**, 4.95 μM for **(+)-1**, and 4.45 μM for **(−)-1**, Table 4], it is evident that the three prodrugs exhibited superior antiproliferative effects. Interestingly, no significant differences in efficacy or potency were observed among the three **(±)-1** derivatives. Furthermore, application of the σ_1_ agonist PTZ, σ_2_ receptor antagonist AC927, or their combination did not perturb the antiproliferative effects of **(±)-1** (Figure 6B), indicating that σ receptors are not the main antiproliferative targets for **(±)-1** in human UM 92-1 cells.

**Table 4:** IC_50_ of **(±)-1**, **(+)-1**, and **(−)-1** on Human 92-1 Cells at 48 h

cmpd	IC_50_ (μM)^a^ ± SD^b^
**(±)-1**	5.5 ± 0.74
**(+)-1**	4.9 ± 0.58
**(−)-1**	4.4 ± 0.68

a IC_50_ values represent absolute estimates calculated with GraphPad Prism 5 for Windows using a nonlinear fit transform sigmoidal dose–response (variable slope). IC_50_ values are averaged from multiple determinations (*n* = 3).

b Values are expressed as mean ± SD of four independent experiments, each conducted in triplicate.

In fact, PTZ and AC927 alone promote, as expected, the proliferation of human UM 92-1 cells, while they are not able to restore the loss of cell viability induced by **(±)-1** (Figure 6B). These results are in agreement with antiproliferative effects in HREC (Table 3 and Figure 2) and suggest that the inhibition of proliferation by prodrugs **(±)-1**, **(+)-1**, and **(−)-1** may reflect the HDACi activity by the VPA molecular component but not the σ receptor signaling by the HP-mII.^29,30^ Indeed, the almost identical antiproliferative potencies exhibited by the three prodrugs would be consistent with the invariable chemistry of VPA constituents but not with the diverse stereochemistry of HP-mII enantiomers, which substantially influences their different binding affinities for σ receptors (Table 2). While excluding σ receptor involvement in the regulation of proliferation by the three prodrug derivatives, present results do not rule out the possible off-target contributions to VPA-dependent inhibition of proliferation, nor elucidate the exact molecular configuration of VPA interaction with the target (as a prodrug or separate, released metabolite).

Regardless, in UM 92-1 cells, superior antiproliferative effects (about 400-fold) by the racemic mixture **(±)-1** and enantiomers with respect to VPA alone (Figure 6A) point toward improved pharmacology provided by the dual-target/dual-function strategy on VPA signaling through HDAC, presumably reflecting a VPA delivery to target sites facilitated by the prodrugs.^45^

**Figure 7:**
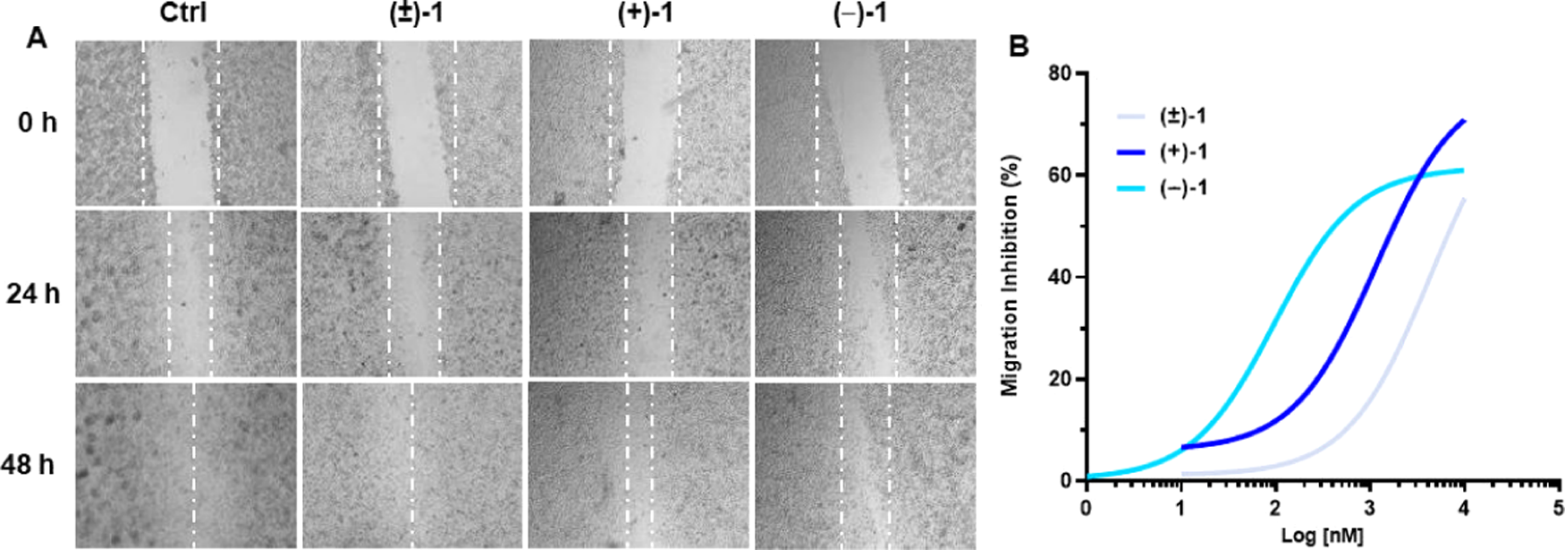
Effect of (±)-1, (+)-1, and (−)-1 on human 92-1 uveal melanoma cell migration. Representative images of the wound healing assay (A). Magnification, 4×. All compounds were used at 3 μM. Ctrl, vehicle control (DMSO). Concentration–response curves of the inhibition of cell migration by the indicated compounds at the 48 h time point (B). Data are shown as % inhibition of cell migration with respect to the vehicle control.

Finally, tumor cell migration was explored with the wound healing assay (Figure 7). **(±)-1**, **(+)-1**, and **(−)-1** inhibited 92-1 cell migration over 48 h time courses (Figure 7A). Remarkably, sigmoid dose–response curves (calculated at 48 h) demonstrated significantly different antimigratory potencies by the three prodrugs (Figure 7B) displaying IC_50_ values of 4.22 μM for **(±)-1**, 1.15 μM for **(+)-1**, and 0.09 μM for **(−)-1**. In particular, the derivative **(−)-1** exerted the highest antimigratory effects compared to the **(+)-**enantiomer (>10-fold) or the racemic mixture **(±)-1** (>40-fold; Figure 7B). These actions closely mirror those observed in HREC migration (Figure 3) and could similarly reflect signaling through σ receptors by the HP-mII molecular component of the prodrugs, without VPA/HDAC involvement. Indeed, their differential potencies in motility inhibition (Figure 7) are aligned with increasing binding affinities for σ_2_ receptors from **(±)-1** to **(+)-1** and **(−)-1** (Table 2), thus implicating the stereochemical diversity of HP-mII enantiomeric components as an underlying molecular mechanism able to regulate cell migration. This hypothesis may also suggest that **(±)-1**, **(+)-1**, and **(−)-1** act predominantly as intact molecular moieties to inhibit cell motility, given the lack of symmetry between observed biological effects (antimigratory activity; Figure 7B) and receptor binding affinities by (±)-HP-mII and single enantiomers (Table 2), which would be released upon prodrug metabolism.

Highest potency (Figure 7B) and σ_2_ selectivity (Figure 3) by the enantiomer **(−)-1**, in turn, would be consistent with its significantly higher binding affinity for σ_2_ receptors than the other two chemical analogues (Table 2) and a presumable biological dominance of σ_2_ over σ_1_ receptors in the context of cell migration. This latter consideration could explain the reciprocal contribution of both σ_1_ and σ_2_ receptors to inhibitory effects on HREC motility by **(±)-1** and **(+)-1** (Figure 3), with weaker σ_2_ binding ligands than **(−)-1** (Table 2). Together, present observations in human 92-1 cells support the dual-target/dual-function strategy (HDACi and σ ligands) underlying the asymmetric synthesis of both enantiomers. Thus, **(+)-1** and **(−)-1** provided potential UM therapeutics with superior, HDAC-mediated antiproliferative activities than single chemical precursors, and enhanced, σ receptor-dependent antimigratory effects compared to the racemic **(±)-1** mixture.

## Conclusions

In this report, the synthesis and pharmacological characterization of the two enantiomers of (±)-HP-mII valproate ester, **(±)-**1, is presented. The results indicate that the three prodrugs exhibit antiangiogenic activity in vitro, comparable to the VEGF-trap AFL but with distinct pharmacological profiles. Indeed, while all compounds induce σ receptor-independent HREC cytotoxicity to a similar extent, HREC migration and tube formation stimulated by VEGF-A are inhibited differently by the three prodrugs through the activation of selective σ receptor signaling. In this context, the enantiomer **(−)-1** displays the highest antimigratory effect on VEGF-A-stimulated HREC by acting as a σ_2_ receptor agonist. Investigations in human UM 92-1 cells demonstrated the potential of the dual-ligand prodrugs as novel anti-UM agents and confirmed molecular pharmacology findings obtained in HREC. In particular, **(±)-1** and its enantiomers induce pharmacologically equivalent cytotoxic effects on 92-1 cells, which are significantly greater than precursor compounds, probably dependent on the HDACi activity by the VPA molecular component but not related to σ receptor signaling. In close agreement with the findings in HREC, all compound prodrugs also significantly inhibit UM 92-1 cell motility with different potencies. Again, the **(−)-1** enantiomer displays the highest antimigratory activity on 92-1 cells, followed by the **(+)-1** stereoisomer and the racemic mixture, pointing toward a presumable involvement of σ receptor pathways and a biological dominance of σ_2_ over σ_1_ signaling in antimigratory effects by the dual-target/dual-function prodrugs. However, the exact molecular mechanisms underlying anti-UM effects by **(−)-1** and its related prodrugs remain unclear and will be the focus of future studies.

On the basis of present findings, **(−)-1** might represent a promising candidate for the development of pharmacological strategies to treat UM patients. In this context, beyond prodrugs with esterification, multiligand drug designs with simultaneous regulation of multiple targets could represent an alternative synthetic approach currently explored in this laboratory.

## Experimental Section

### Chemistry

Reagent grade chemicals were purchased from Merck KGaA (Darmstadt, Germany) and TCI Europe–Tokyo chemical industry (Tokyo, Japan) and were used without further purification. All reactions involving air-sensitive reagents were performed under N_2_ in oven-dried glassware using the syringe-septum cap technique. The reactions were monitored by thin-layer chromatography (TLC) performed on silica gel Merck 60 F_254_-coated aluminum plates; the spots were visualized by UV light (λ = 254 nm) or iodine chamber. Melting points were determined on a Büchi B-450 apparatus in glass capillary tubes and are uncorrected. Optical rotations were taken at 20 °C with a PerkinElmer 241 polarimeter (Llantrisant, U.K.). Flash chromatography purification was performed on a Merck silica gel 60 (40–63 μm; 230–400 mesh) stationary phase. Nuclear magnetic resonance spectra (^1^H NMR and ^13^C NMR recorded at 500 MHz) were obtained on Varian INOVA spectrometers using CDCl_3_, D_2_O, and DMSO-*d*_6_ with 0.03% tetramethylsilane (TMS) as the internal standard. Chemical shifts (δ) are given in parts per million (ppm) and coupling constants (*J*) in Hertz (Hz). Signal multiplicities are characterized as s = singlet, d = doublet, t = triplet, q = quartet, m = multiplet, and br = broad. High-resolution mass spectrometry (HR-MS) was performed using an Orbitrap Fusion Tribrid mass spectrometer (Thermo Scientific). Compounds at the concentration of 10 μg/mL were dissolved in ACN/H_2_O (80/20) + 0.1% formic acid. Parameters: polarity (positive). Full scan mass was analyzed at a resolution of 120 000. Purities of all compounds reached at least 95% as determined by microanalysis (C, H, N) that was performed on a Carlo Erba instrument model E1110; all of the results agreed within ±0.4% of the theoretical values. Compound nomenclatures were generated with ChemBioDraw Ultra version 16.0.0.82.

### Synthesis of Compounds **(+)-3**, **(−)-3**, **(+)-4**, and **(−)-4**

All compounds were synthesized as reported in the literature.^33^ General procedure and analytical and spectral data are reported in the Supporting Information.

### General Procedure for the Synthesis of Compounds **(+)-1** and **(−)-1**

To a solution of **(+)-4** or **(−)-4** (0.5 mmol) and triethylamine (TEA) (1 mmol) in anhydrous THF (6 mL) was added 2-propylpentanoyl chloride (2 mmol) at 0 °C and under stirring. The reaction was left at room temperature for 24 h under N_2_. The reaction mixture was quenched with 15 mL of water and stirred for 30 min. To the mixture was added CH_2_Cl_2_, and the organic phase separated and washed with a solution of 4% NaHCO_3_ (3 × 25 mL). The organic layers were dried over anhydrous Na_2_SO_4_, filtered, and evaporated. The crude was purified by flash chromatography (1:9 MeOH/CH_2_Cl_2_) to obtain the final products **(+)-1** and **(−)-1** as colorless oil. Both enantiomers were transformed into oxalate salts.

#### (*R*)-(+)-4-[(4-Chlorophenyl)-4-hydroxypiperidin-1-yl]-1-(4-fluorophenyl)butyl-2-propylpentanoate [(*R*)-(+)-MRJF22 Oxalate, **(+)-1**]

According to the general procedure, compound **(+)-1** was prepared by reacting 2-propylpentanoyl chloride (0.195 g, 1.2 mmol) and compound **(+)-4** (0.113 g, 0.3 mmol). Yield: 0.095 g (63%), white solid. Mp: 159–164 °C. [α]_D_^20^= +24.8° (*c*1.0, CHCl_3_). 92% ee ^1^H NMR (500 MHz, DMSO-*d*_6_): δ 7.51–7.45 (m, 2H), 7.44–7.38 (m, 4H), 7.20 (t, *J* = 10.0 Hz, 2H), 5.72 (t, *J* = 5.0 Hz, 1H), 3.33–3.19 (m, 2H), 3.18–2.99 (m, 4H), 2.37 (m, 1H), 2.22–2.07 (m, 2H), 1.91–1.85 (m, 1H), 1.81–1.53 (m, 5H), 1.52–1.43 (m, 2H), 1.42–1.43 (m, 2H), 1.23–1.09 (m, 4H), 0.84 (t, *J* = 5.0 Hz, 3H), 0.79 (t, *J* = 5.0 Hz, 3H). ^13^C NMR (125 MHz, DMSO-*d*_6_): δ 174.6, 164.6 (*J*_CF_ = 213.75 Hz), 146.9, 136.5, 131.4, 128.5, 128.4 (*J*_CF_ = 7.5 Hz), 126.6, 115.2 (*J*_CF_ = 21.25 Hz), 73.8, 67.9, 47.9, 48.9, 44.4, 37.9, 35.04, 34.03, 33.9, 20.04, 19.9, 19.8, 13.7. HR-MS: *m*/*z* [M + H]^+^ calcd for C_29_H_40_ClFNO_3_: 504.2681, found: 504.2663. Anal. calcd for C_31_H_41_ClFNO_7_: C, 74.17; H, 8.58; N, 2.98. Found: C, 74.22; H, 8.61; N, 2.98.

#### (*S*)-(−)-4-[(4-Chlorophenyl)-4-hydroxypiperidin-1-yl]-1-(4-fluorophenyl)butyl-2-propylpentanoate [(*S*)-(−)-MRJF22 Oxalate, **(−)-1**]

According to the general procedure, compound **(−)-1** was prepared by reacting 2-propylpentanoyl chloride (0.325 g, 2.0 mmol) and compound **(−)-4** (0.189 g, 0.5 mmol). Yield: 0.252 g (100%), white solid. Mp: 158–160 °C. [α]_*D*_^20^= −26.0° (*c*1.0, CHCl_3_). 95.4% ee ^1^H NMR (500 MHz, DMSO-*d*_6_): δ 7.50–7.46 (m, 2H), 7.44–7.38 (m, 4H), 7.20 (t, *J* = 10.0 Hz, 2H), 5.72 (t, *J* = 5.0 Hz, 1H), 3.33–3.19 (m, 2H), 3.18–2.99 (m, 4H), 2.37 (m, 1H), 2.22–2.07 (m, 2H), 1.91–1.85 (m, 1H), 1.81–1.53 (m, 5H), 1.52–1.43 (m, 2H), 1.42–1.43 (m, 2H), 1.23–1.09 (m, 4H), 0.84 (t, *J* = 5.0 Hz, 3H), 0.79 (t, *J* = 5.0 Hz, 3H). ^13^C NMR (125 MHz, DMSO-*d*_6_): δ 174.4, 162.4 (*J*_CF_ = 212.5 Hz), 146.7, 136.4, 131.3, 128.3 (*J*_CF_ = 8.7 Hz), 120.9, 127.9, 126.5, 115.0 (*J*_CF_ = 21.25 Hz), 73.6, 67.7, 44.3, 33.9, 33.8, 32.6, 19.9, 19.8, 19.7, 13.6, 13.5. HR-MS: *m*/*z* [M + H]^+^ calcd for C_29_H_40_ClFNO_3_: 504.2681, found: 504.2663. Anal. calcd for C_31_H_41_ClFNO_7_: C, 74.17; H, 8.58; N, 2.98. Found: C, 74.22; H, 8.61; N, 2.98.

### Chromatographic Conditions

The liquid chromatography system was an Agilent 1260 Infinity II HPLC (Agilent, Santa Clara, CA) consisting of a 1260 Infinity II Quaternary Pump (model G7111A), 1260 Infinity II autosampler (model G7129A), a 1260 Infinity II Multicolumn Thermostat (model G7116A), and a 1260 Infinity II Diode Array Detector (model G7115A). Data were acquired and integrated using the software Agilent OpenLab CDS LC ChemStation. The separation was performed using a Poroshell 120 EC-C18 (150 × 4.6 mm i.d., particle size 4 μm; Agilent, Santa Clara), maintained at 20 °C. The samples were run using a mixture of water (A) and acetonitrile (B) enriched with trifluoroacetic acid (0.1% v/v). The gradient used was from 80% A to 100% B over 10 min. The flow rate was 0.8 mL/min. The UV detector was set at a length of 254 nm. Enantioselective HPLC analyses were performed using the same above-described conditions except for the stationary phase, which was a Chiralcel OJ[-RH] column (150 × 4.6 mm, 5 μm).

### Kinetics of Chemical Hydrolysis

A 0.02 M phosphate buffer (PBS, pH 7.4) and a 0.02 M hydrochloric buffer (pH 1.3) containing 0.1% (v/v) Cremophor ELP, was used to evaluate chemical stabilities at physiological pHs. The reaction was initiated by adding 1 mL of 10^–4^ M stock solution (in acetonitrile) of the compound to 10 mL of thermostated (37 ± 0.5 °C) buffer solution. At established time points, the samples (20 μL) were withdrawn and analyzed by HPLC. Pseudo-first-order rate constants (*k*_obs_) for the hydrolysis of the compounds were then calculated considering the slopes of the linear plots of log (% residual compound) against time. The analyses were run in triplicate, and the mean values of the rate constants were calculated.

### Kinetics of Enzymatic Hydrolysis

Human plasma was purchased from 3H Biomedical (Uppsala, Sweden, Europe). Plasma aliquots (4 mL) were diluted with 0.02 M PBS (pH 7.4) to obtain a final volume of 5 mL containing 80% plasma. Studies were performed at 37 ± 0.5 °C using a shaking bath. Each experiment was started by adding a 10^–4^ M drug stock solution (200 μL) to the preheated plasma. Hundred microliters of the thermostated medium was taken at various times, treated with cold methanol (500 μL) to precipitate plasma proteins, and centrifugated (5 min at 5000*g*). The supernatant was analyzed by HPLC to quantify the amount of the residual intact compound.

### Receptor Binding Studies

The σ_1_ and σ_2_ receptor binding studies were performed according to the literature.^46,47^ Briefly, for the σ_1_ receptor binding assay, guinea pig brain membranes (400 μL, 500 μg protein) were incubated for 150 min at 37 °C with 3 nM of the radiolabeled ligand [^3^H]-(+)-pentazocine (45 Ci/mmol) and increasing concentrations of tested compounds in 50 mM Tris-HCl (pH 7.4) to a total volume of 1 mL. Nonspecific binding was assessed in the presence of 10 μM unlabeled haloperidol. Moreover, σ_2_ receptor binding assays were made according to the following protocol: the guinea pig brain membranes (300 μL, 360 μg protein) were incubated for 120 min at room temperature with 3 nM [^3^H]-DTG (31 Ci/mmol) in the presence of 0.4 mM radiolabeled ligand (+)-SKF10,047 to block the σ_1_ sites. The incubation was performed in 50 mM Tris-HCl (pH 8.0) to a total volume of 0.5 mL with increasing concentrations of each test compound. Nonspecific binding was evaluated in the presence of 5 μM DTG.

Each sample was filtered through Whatman GF/B glass fiber filters, which were presoaked for 1 h in a 0.5% poly(ethylenimine) solution, using a Millipore filter apparatus. Filters were washed twice with 4 mL of ice-cold buffer. Radioactivity was counted in 4 mL of “Ultima Gold MV” in a 1414 WinSpectral PerkinElmer Wallac or Beckman LS6500 scintillation counter. Inhibition constants (*K*_i_ values) were calculated using the EBDA/LIGAND program purchased from Elsevier/Biosoft.

### Cell Cultures

Primary HREC were purchased from Innoprot (Elexalde Derio, Spain) and were fed with culture EC medium, supplemented with 5% fetal bovine serum (FBS), 1% endothelial cell growth supplement (ECGS), 100 U/mL penicillin, and 100 μg/mL streptomycin provided by Innoprot. The cells were plated in T25 culture flasks (Costar; Corning, New York, NY), precoated with fibronectin (Innoprot) for 1 h at 37 °C. Further, human uveal melanoma (UM) 92-1 cell line was purchased from the Cell Factory-IST (Genoa, Italy). Human UM cell line 92-1 (passages 2–15) were maintained at 37 °C (5% CO_2_) in RPMI-1640 medium, containing 10% fetal bovine serum (FBS), 2 mM l-glutamine, 100 units/mL penicillin, and 100 μg/mL streptomycin.^48^ Breast cancer cell line MCF-7, obtained from the American Type Culture Collection (ATCC; Manassas, VA), was maintained at 37 °C (5% CO_2_) in Eagle’s Minimum Essential Medium, containing 10% FBS, 2 mM l-glutamine, 100 units/mL penicillin, 100 μg/mL streptomycin, and 0.01 mg/mL human recombinant insulin. All cell media and reagents were from Euroclone S.p.A. (Pero, Milan, Italy). (+)-Pentazocine (Italian Minister of Health permit to produce and use SP/072 05/04/2019) and AC927 were prepared according to published methods.^49,50^

### MTT Assay

For cell viability assays, the 3-[4,5–dimethylthiazol-2-yl]-2,5-diphenyltetrazolium bromide (MTT assay, Chemicon, Temecula, CA) was used. The cells were seeded in 96-well plates at a density of 1.5 × 10^4^ cells/well and were incubated overnight at 37 °C before the experiment. Afterward, the cells were treated for 24, 48, and 72 h in the presence of different concentrations (1.0, 2.5, 5.0, 10.0, 20.0 μM) of **(±)-1**, **(+)-1,** or **(−)-1**. After the treatments, the cells were incubated with MTT (5 mg/mL) for 3 h, and then 100 μL of dimethyl sulfoxide was added, and the absorbance was read in a plate reader (Synergy 2-BioTek) with a wavelength of 570 nm.

### Cell Proliferation

HREC and human 92-1 cell (seeded 4 × 10^3^ and grown for 24 h at optimum culture conditions) proliferation was measured using crystal violet staining after treatment in 96-well plates with the indicated concentrations of chemicals for an additional 48 h. Control cells received an equal volume of vehicle (DMSO). At the end of the treatment, each well was washed with phosphate-buffered saline (PBS). Following this, the cells were fixed (in 4% paraformaldehyde) and stained with crystal violet solution (0.5% in 20% methanol for HREC or 1% aqueous solution for 92-1 cells). Subsequently, the plate was washed with water and left to dry. Crystal violet staining was evaluated by measuring the absorbance at 570 nm or 590 nm, after crystal violet extraction with 10% acetic acid (at room temperature for 10 min), with a microplate reader with the plate reader (Synergy 2-BioTek). For quantification, each assay was carried out in triplicate.

### Wound Healing Assay

HREC and 92-1 cells (2.5 × 10^5^ cells/well) were seeded into 24 well plates and grown to confluency. Confluent cell monolayers were scratched with a p200 pipet tip to create 1 mm wide wounds. After washing (three times) with PBS to remove cell debris, wounded monolayers were incubated for 48 h (37 °C) in the complete medium in the presence of the indicated treatments. HREC were treated for 48 h in the presence of **(±)-1**, **(+)-1**, or **(−)-1** with or without VEGF-A (80 ng/mL), AC927, and PTZ. 92-1 cells were treated for 48 h in the presence of **(±)-1**, **(+)-1,** or **(−)-1**. Wound closure was monitored by photographs at 40X using a phase-contrast microscope in each culture condition and at each time point (0, 24, and 48 h for HREC and 0, 6, 24, 30, and 48 h for 92-1 cells). The numbers of cells toward the wounds were counted using ImageJ software (ImageJ 1.50e, National Institutes of Health, NIH, Bethesda, MD) and were quantified by measuring the distance traveled over time by both cell fronts into the wound area.

### Tube Formation Assay

The tube formation assay was analyzed in vitro in BD Matrigel, following the manufacturer’s instructions (BD, Bedford). In brief, 96-well plates were coated with 50 μL of Matrigel and allowed to solidify at 37 °C for 2 h. HREC were seeded at 1.5 × 10^4^ cells/well in 100 μL of medium with **(±)-1**, **(+)-1,** or **(−)-1** in the presence or absence of VEGF-A (80 ng/mL), AC927, and PTZ. After 8 h of incubation, tubelike structures were photographed at 10× magnification using an inverted microscope (Leica DM IRB) equipped with a CCD camera. Tube formations were quantified with ImageJ software (NIH, Bethesda, MD).

### Total RNA and RT-PCR

Total RNA was extracted from cell cultures employing Direct-zol RNA Miniprep (Zymo Research, California) according to manufacturer’s instructions and redissolved in 30 μL of RNase-free water. RNA concentrations and purity were estimated by optical density at 260 and 280 nm. The first-strand cDNA was reversely transcribed using the High-Capacity cDNA Reverse Transcription Kit (Applied Biosystems, California) in a 20 μL reaction volume. Aliquots of cDNA were amplified using specific primers for the σ_1_ receptor (SIGMAR1, F: 5′-GTGAGGTCTTCTACCCAG-3′ and R: 5′-GAAGAGGGTGAGGAAGTC-3′), σ_2_ receptor (TMEM97, F:5′-CCTGGTTTAAGTCCTTTCTG-3′; R:CTCAAACAGAAATGTGGAGAG-3′), and the positive control 45S Ribosomal pre-RNA (F: CGCGCTCTACCTTACCTACCT and R:CGTCGGCATGTATTAGCTCT), producing three specific amplification products of 173 bp, 173 bp, and 199 bp, respectively. PCR reactions were carried out employing the Wonder Taq kit (Euroclone). PCR parameters were as follows: initial denaturing, 95 °C for 3 min; 35 cycles of denaturing at 95 °C for 15 s; annealing at 54 °C (SIGMAR1), 56 °C (TMEM97), or 59 °C (45S Ribosomal pre-RNA) for 15 s and extension at 72 °C for 30 s; and final extension step, 72 °C for 7 min. Primers for RT-PCR were purchased from Sigma Aldrich (St. Louis, Missouri).

### Statistical Analysis

All results are reported as mean ± SEM from at least two or three independent experiments (*n* = 2 or 3) performed at least in duplicate or triplicate. The results were analyzed using one-way ANOVA followed by Tukey–Kramer multiple comparisons test; differences between groups were considered significant for *p*-value < 0.05.

## Supporting Information Available

Detailed procedures for the preparation of compounds **(+)-3**, **(−)-3**, **(+)-4,** and **(−)-4**; HPLC chromatograms of compounds **(±)-1**, **(+)-1**, or **(−)-1**; the MTT assay concentration–response curve on HREC; representative images of scratched cells treated with VEGF-A and the wound healing assay on HREC of compounds **(±)-1**, **(+)-1**, or **(−)-1**; ^1^H and ^13^C NMR spectra and HRMS spectra (PDF)
Molecular formula strings (CSV)

## Abbreviations

AFL: aflibercept
BRB: blood-retinal barrier
DIP-Cl: diisopinocampheylchloroborane
FBS: fetal bovine serum
HDACi: histone deacetylase inhibitor
hERG: human voltage-dependent K^+^ channel
HP: haloperidol
HP-mII: haloperidol metabolite II
HREC: human retinal endothelial cell
(+)-PTZ: (+)-pentazocine
RT-PCR: reverse transcription polymerase chain reaction
TEA: triethylamine
TLC: thin-layer chromatography
UM: uveal melanoma
VEGF-A: vascular endothelial growth factor A
VPA: valproic acid
