# Supplementary material for: Haloperidol Metabolite II Valproate Ester (S)-(−)-MRJF22: Preliminary Studies as a Potential Multifunctional Agent Against Uveal Melanoma
Source: J Med Chem. 2021 Sep 3;64(18):13622–32. doi: 10.1021/acs.jmedchem.1c00995 (PMC8474110; doi:10.1021/acs.jmedchem.1c00995)
Supplement: Supplementary file 1 — jm1c00995_si_001.pdf [file jm1c00995_si_001.pdf]

Supporting information

Haloperidol Metabolite II Valproate Ester (*S*)-(−)-MRJF22: Preliminary Studies as Potential Multifunctional Agent Against Uveal Melanoma

Carla Barbaraci,[†,#,§] Giovanni Giurdanella,[‡,§] Claudia Giovanna Leotta,[#] Anna Longo,[‡] Emanuele Amata,[†] Maria Dichiara,[†] Lorella Pasquinucci,[†] Rita Turnaturi,[†] Orazio Prezzavento,[†] Ivana Cacciatore,[ϕ] Elisa Zuccarello,[Σ] Gabriella Lupo,[‡] Giovanni Mario Pitari,*,[#] Carmelina Daniela Anfuso,*,[‡] Agostino Marrazzo*,[†]

[†]Department of Drug and Health Sciences, University of Catania, Viale A. Doria 6, 95125 Catania, Italy

[‡]Department of Biomedical and Biotechnological Sciences, School of Medicine, University of Catania, Via S. Sofia 97, 95123 Catania, Italy

[#]Vera Salus Ricerca S.r.l, Via Sigmund Freud 62/B, 96100 Siracusa, Italy

[φ]Department of Pharmacy, "G. D'Annunzio" University of Chieti-Pescara, Via dei Vestini 31, 66100 Chieti Scalo, Italy

[Σ]Taub Institute for Research on Alzheimer's Disease and the Aging Brain, Columbia University, New York, New York 10032, United States.

Corresponding Author

*Agostino Marrazzo: phone, (+39) 095 7834250; email, marrazzo@unict.it

* Carmelina Daniela Anfuso: phone: (+39) 095 4781170; email: daniela.anfuso@unict.it

* Giovanni Mario Pitari: phone, (+39) 0931 1987360; email, giovanni.pitari@verasalusricerca.it

Table of contents

Chemistry	S3
Figure S1	S5
Figure S2	S6
Figure S3	S7
Figure S4	S8
Figure S5	S9
NMR spectra	S10
HR-MS spectra	S12

Chemistry

General procedure for the synthesis of compounds (–)-3 and (+)-3. A solution of 4-chloro-1-(4-fluorophenyl)butan-1-one (**2**, 1 mmol) in dry THF was added dropwise to a solution of (+) or (−)-diisopinocanphenylchloroborane (DIP-Cl) in THF dry at −25 °C. The mixture was stirred at this temperature for 16 h under N_2. The solvent was removed under vacuum and the residue was taken up into diethyl ether. To this solution was carefully added diethanolamine (DEA) and let stirred under N_2 overnight. The white salt formed was separated by filtration through Celite® and the filtrate was concentred. The reaction mixture was quenched with a solution of HCl 1M and the aqueous phase extracted with CH_2Cl_2 (3 x 50 mL). The organic phase was dried over anhydrous Na_2SO_4, filtered and evaporated. The crude product was purified by flash chromatography (1:9 EtOAc/Cy) to obtain the desired products.

(*R*)-(+)-4-chloro-1-(4-fluorophenyl)butan-1-ol [(+)-3]. Compound **(+)-3** was prepared according to the general procedure using **2** (0.400 g, 2.0 mmol) and (+)-DIP-Cl (2.570 g, 8.0 mmol). Yield: 0.385 g (95%), white solid. $[\alpha]_D^{20}$= +37° (*c*1.0, $CHCl_3$). ^{1}H NMR (500 MHz, $CDCl_3$) δ 7.42−7.28 (m, 2H), 7.07−6.99 (m, 2H), 4.70 (t, *J* = 6.0 Hz, 1H), 3.58−3.52 (m, 2H), 2.57−2.30 (m, 2H), 2.12−1.63 (m, 3H). ^{13}C NMR (500 MHz, $CDCl_3$) δ 164.7 (J_{CF} = 325.0 Hz), 141.2, 131.1 (J_{CF} = 8.5 Hz), 115.7 (J_{CF} = 35.0 Hz), 72.1, 44.2, 37.2, 24.3. Anal. Calcd for $C_{10}H_{12}ClFO$: C, 59.27; H, 5.97; N, 17.49. Found: C, 59.50; H, 6.07; N, 17.58.

(*S*)-(–)-4-chloro-1-(4-fluorophenyl)butan-1-ol [(–)-3]. Compound **(–)-3** was prepared according to the general procedure using **2** (0.500 g, 2.0 mmol) and (-)-DIP-Cl (0.600 g, 12.0 mmol). Yield: 0.340 g (84%), white solid. $[\alpha]_D^{20}$= –41° (*c*1.2, $CHCl_3$). ^{1}H NMR (500 MHz, $CDCl_3$) δ 7.42−7.28 (m, 2H), 7.07−6.99 (m, 2H), 4.70 (t, *J* = 6.0 Hz, 1H), 3.58−3.52 (m, 2H), 2.57−2.30 (m, 2H), 2.12−1.63 (m, 3H). ^{13}C NMR (125 MHz, $CDCl_3$) δ 164.7 (J_{CF} = 325.0 Hz), 141.2, 131.1 (J_{CF} = 8.5 Hz), 115.7 (J_{CF} = 35.0 Hz), 72.1, 44.2, 37.2, 24.3. Anal. Calcd for $C_{10}H_{12}ClFO$: C, 59.27; H, 5.97; N, 17.49. Found: C, 59.45; H, 6.07; N, 17.79.

General procedure for the synthesis of compounds (+)-4 and (−)-4. Both compounds were synthesized as reported in literature.[33] To a solution of 4-(4-chlorophenyl)hydroxypiperidine (4 mmol) and $KHCO_3$ (4 mmol) in dry DMF (12 mL) was added dropwise a solution **(–)-3** or **(+)-3** (1 mmol). The reaction mixture was heated to 80 °C for 24 h. The solvent was removed under vacuum and the residue was dissolved in $CHCl_3$ and washed with $NaHCO_3$ (3 x 25 mL). The organic layer was dried over anhydrous Na_2SO_4, filtered and evaporated. The crude product was purified by flash chromatography (1:9 MeOH/CH_2Cl_2) to obtain the desired products.

(*R*)-(+)-4-(4-chlorophenyl)-1-[4(4-fluorophenyl)-4-hidroxybutyl]piperidin-4-ol [(*R*)-(+)-HP-mII, (+)-4]. According to general procedure, compound **(+)-4** was prepared by reacting 4-(4-chlorophenyl)hydroxypiperidine (1.140 g, 5.0 mmol) and compound **(+)-3** (0.362 g, 1.79 mmol). Yield 0.189 g (28%), white solid. Mp: 145-146.5 °C; of, $[\alpha]_D^{20}$= +66° (*c*1.0, $CHCl_3$). ^{1}H NMR (500 MHz, $CDCl_3$) δ 7.47−7.27 (m, 6H), 7.05−6.95(m, 2H), 4.65−4.62 (m, 1H), 3.05−2.99 (m, 1H), 2.85−2,79 (m, 1H), 2,67−2,42 (m, 4H), 2.28−2.12 (m, 2H), 2.01−1.79 (m, 8H). ^{13}C NMR (125 MHz, $CDCl_3$) δ 164.7 (J_{CF} = 325.5 Hz), 145.7, 138.3, 130.4, 130.4 (J_{CF} = 8.5 Hz), 120.9, 115.8 (J_{CF} = 35.5 Hz), 73.0, 71.1, 55.7, 48.1, 39.6, 39.4, 24.8. Anal. Calcd for $C_{21}H_{25}ClFNO_2$: C, 65.19; H, 6.77; N, 3.62. Found: C, 65.37; H, 7.02; N, 3.20.

(*S*)-(–)-4-(4-chlorophenyl)-1-[4(4-fluorophenyl)-4-hidroxybutyl]piperidin-4-ol [(*S*)-(–)-HP-mII, (–)-4]. According to general procedure, compound **(–)-4** was prepared by reacting 4-(4-chlorophenyl)hydroxypiperidine (0.865 g, 4.0 mmol) and compound **(–)-3** (0.206 g, 1.0 mmol). Yield 0.291 g (77%), white solid. Mp: 145−146 °C. $[\alpha]_D^{20}$=–65° (*c*1.0, $CHCl_3$). ^{1}H NMR (500 MHz, $CDCl_3$) δ 7.47−6.83 (m, 8H), 4.65−4.62 (m, 1H), 3.05–2.99 (m, 1H), 2.85−2,79 (m, 1H), 2,67−2,42 (m, 4H), 2.31−2.23 (m, 2H), 2.10−1.50 (m, 8H). ^{13}C NMR (125 MHz, $CDCl_3$) δ 164.67 (J_{CF} = 325.5 Hz), 145.7, 138.3, 130.4, 130.4 (J_{CF} = 8.5 Hz), 120.9, 115.8 (J_{CF} = 35.5 Hz), 73.0, 71.1, 55.7, 48.1, 39.6, 39.4, 24.8. Anal. Calcd for $C_{21}H_{25}ClFNO_2$: C, 65.19; H, 6.77; N, 3.62. Found: C, 64.97; H, 6.47; N, 3.99.

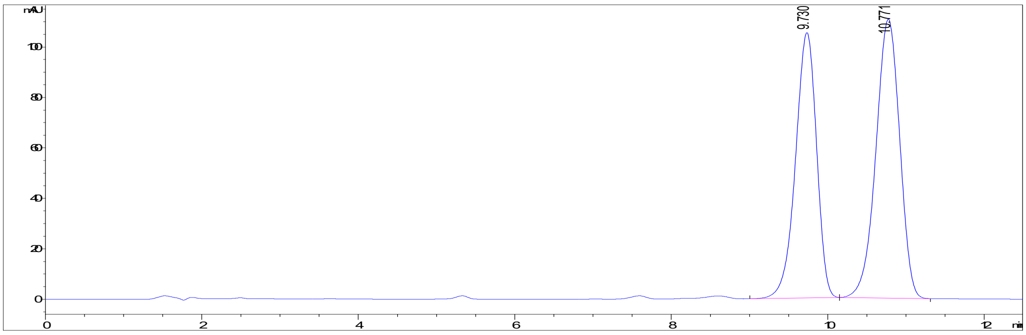

Compound	Retention time	Area%
(*R*)-(+)-MRJF22	9.7	45.7
(*S*)-(−)-MRJF22	10.7	54.3

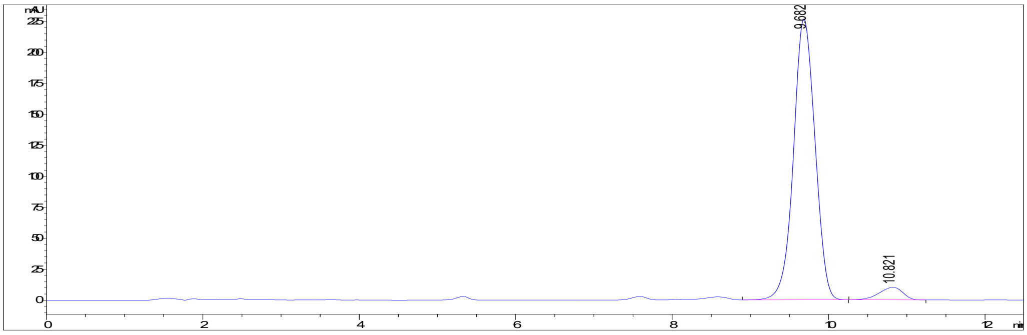

Compound	Retention time	Area%	ee%
(*R*)-(+)-MRJF22	9.7	95.3	92
(*S*)-(−)-MRJF22	10.7	4	

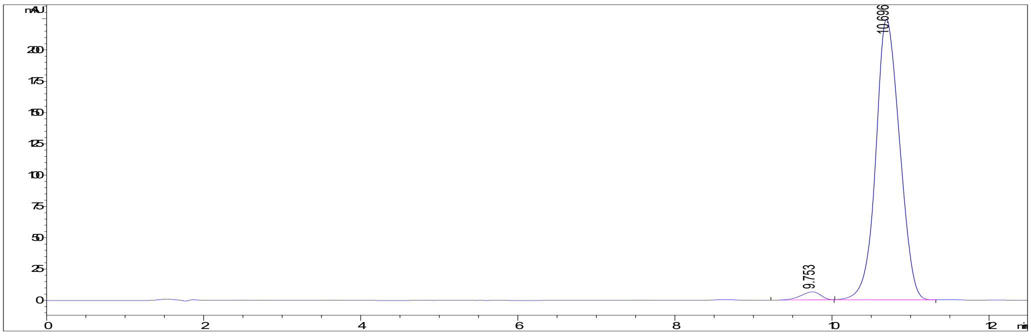

Compound	Retention time	Area%	ee%
(*R*)-(+)-MRJF22	9.7	2.3	95.4
(*S*)-(−)-MRJF22	10.7	97.7	

Figure S1. HPLC chromatograms of **(±)-MRJF22**, ***(R)*-(+)-MRJF22**, and ***(S)*-(−)-MRJF22**.

Effects of (−)-1 and (+)-1 on HREC viability at three different time points.

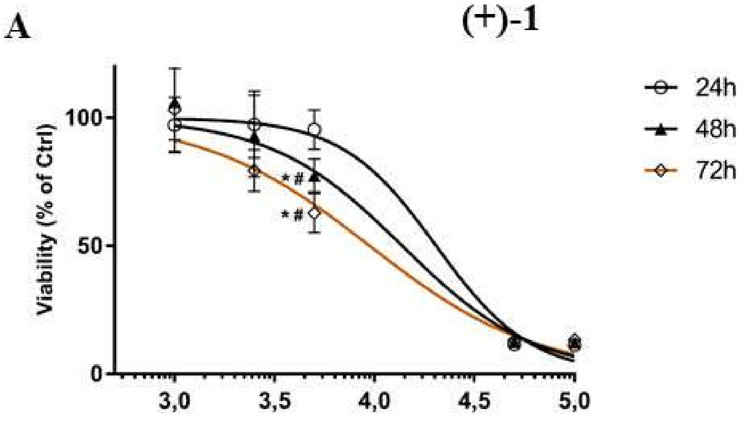

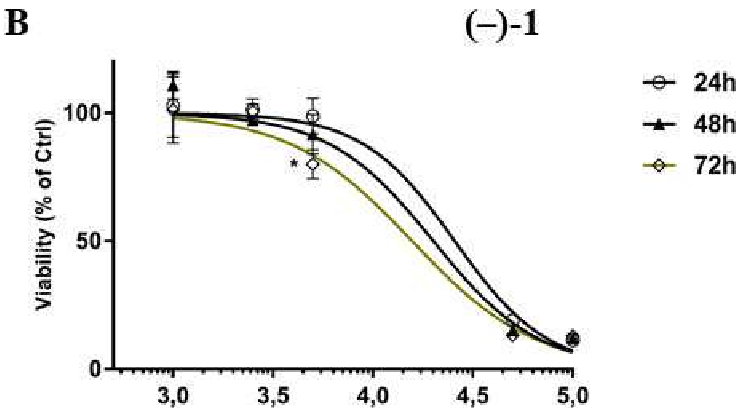

Figure S2. MTT values obtained in cells treated with increasing concentration (1.0, 2.5, 5.0, 10.0 and 20.0 μM) of **(–)-1** (A) and **(+)-1** (B) for 24, 48 and 72 h. Data are expressed as a mean ± SEM of three independent experiments, each involving three different wells per condition. Statistical analysis was performed using two-way ANOVA, followed by Tukey's test. $^{*}p$ <0.05 *vs* control; $^{\#}p$ <0.05 *vs* 24 h 5 μM.

Wound healing assay: effects of (±)-1 on VEGF-A stimulated HREC

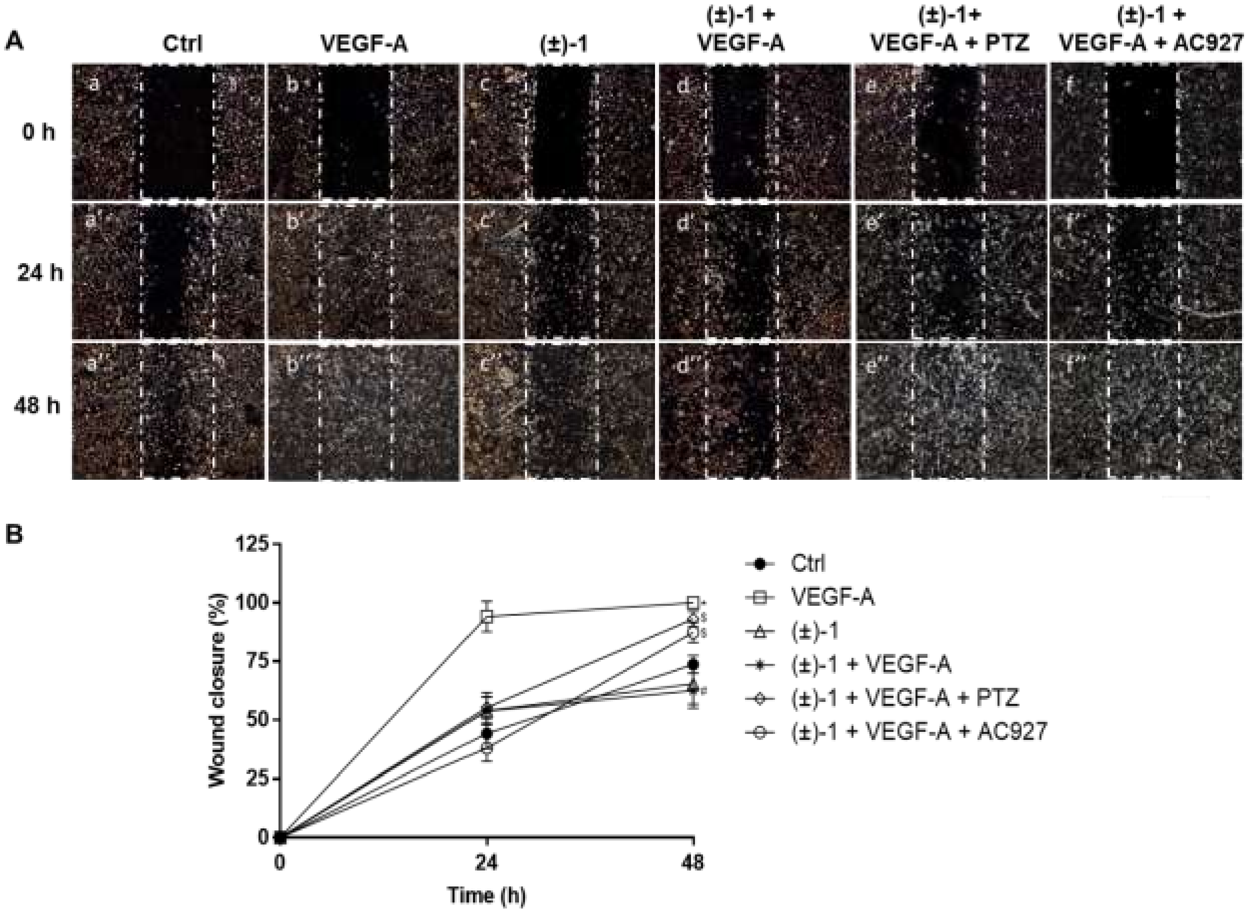

Figure S3. A) Representative images of scratched cells treated with VEGF-A (80 ng/mL; b, b' and b''), 5 μM of **(±)-1** (c, c' and c''), VEGF-A plus 5 μM of **(±)-1** (d, d' and d''), VEGF-A plus 5 μM **(±)-1** plus 2 μM of Pentazocine (PTZ, e, e' and e''), VEGF-A plus 5 μM of **(±)-1** plus 2 μM of AC927 (f, f' and f''). Untreated cells were considered as control samples (Ctrl; a, a' and a''). Images show cells at the starting points (0 h after scratch: a, b, c, d, e and f), after 24 h (a', b', c', d', e' and f') and 48 h (a'', b'', c'', d'', e'' and f'') from the starting of the assays. B) Wound closure percentage with **(±)-1** was quantified by ImageJ software. Ctrl, vehicle control (DMSO). Values are expressed as a mean ± SEM of three independent experiments, each involving three different wells per condition. Statistical analysis was performed using one-way ANOVA, followed by Tukey's test. $^{*}p$ <0.05 *vs* Ctrl; $^{\#}p$ <0.05 *vs* VEGF-A; $^{\S}p$ <0.05 *vs* the same conditions without agonist or antagonist.

Wound healing assay: effects of (+)-1 on VEGF-A stimulated HREC

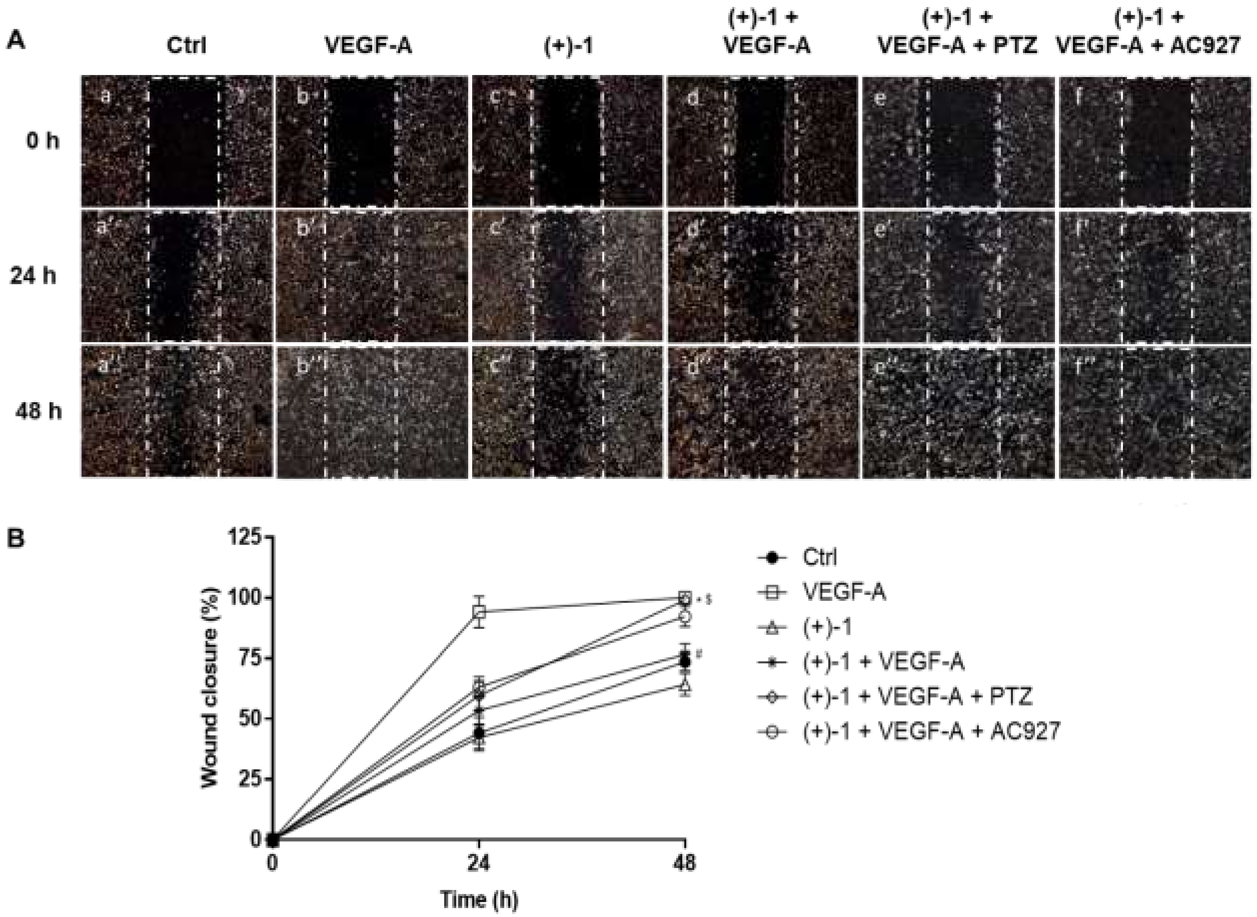

Figure S4. A) Representative images of scratched cells treated with VEGF-A (80 ng/mL; b, b' and b''), 5 µM of **(+)-1** (c, c' and c''), VEGF-A plus 5 µM of **(+)-1** (d, d' and d''), VEGF-A plus 5 µM **(+)-1** plus 2 µM of PTZ (e, e' and e''), VEGF-A plus 5 µM of **(+)-1** plus 2 µM of AC927 (f, f' and f''). Untreated cells were considered as control samples (Ctrl; a, a' and a''). Images show cells at the starting points (0 h after scratch: a, b, c, d, e and f), after 24 h (a', b', c', d', e' and f') and 48 h (a'', b'', c'', d'', e'' and f'') from the starting of the assays. B) Wound closure percentage with **(+)-1** was quantified by ImageJ software. Ctrl, vehicle control (DMSO). Values are expressed as a mean ± SEM of three independent experiments, each involving three different wells per condition. Statistical analysis was performed using one-way ANOVA, followed by Tukey's test. $^{*}p$ <0.05 *vs* Ctrl; $^{\#}p$ <0.05 *vs* VEGF-A; $^{\S}p$ <0.05 *vs* the same conditions without agonist or antagonist.

Wound healing assay: effects of (–)-1 on VEGF-A stimulated HREC.

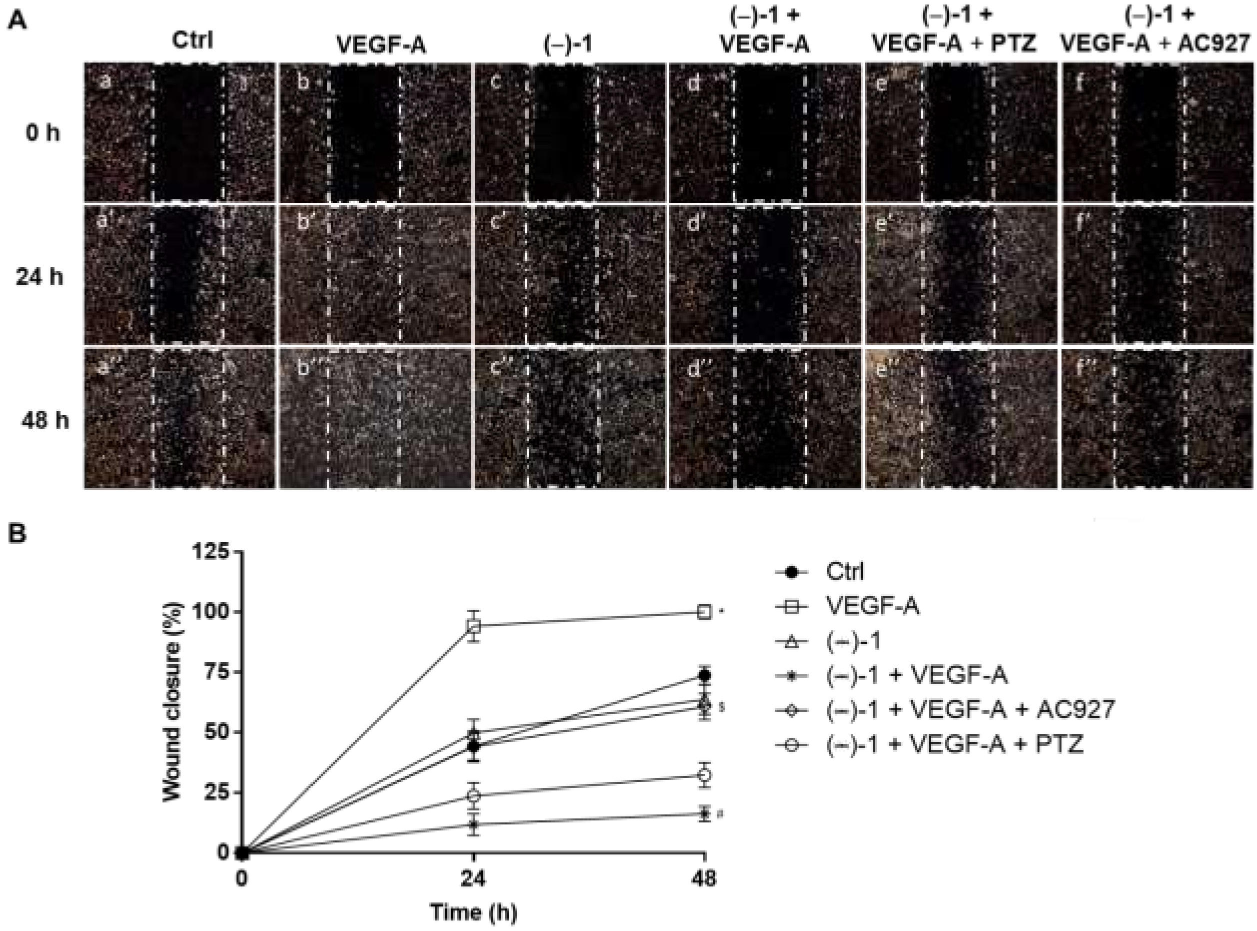

Figure S5. A) Representative images of scratched cells treated with VEGF-A (80 ng/mL; b, b' and b''), 5 μM of **(–)-1** (c, c' and c''), VEGF-A plus 5 μM of **(–)-1** (d, d' and d''), VEGF-A plus 5 μM **(–)-1** plus 2 μM of PTZ (e, e' and e''), VEGF-A plus 5 μM of **(–)-1** plus 2 μM of AC927 (f, f' and f''). Untreated cells were considered as control samples (Ctrl; a, a' and a''). Images show cells at the starting points (0 h after scratch: a, b, c, d, e and f), after 24 h (a', b', c', d', e' and f') and 48 h (a'', b'', c'', d'', e'' and f'') from the starting of the assays. B) Wound closure percentage with **(–)-1** was quantified by ImageJ software. Ctrl, vehicle control (DMSO). Values are expressed as a mean ± SEM of three independent experiments, each involving three different wells per condition. Statistical analysis was performed using one-way ANOVA, followed by Tukey's test. $^{*}p$ <0.05 *vs* Ctrl; $^{\#}p$ <0.05 *vs* VEGF-A; $^{\S}p$ <0.05 *vs* the same conditions without agonist or antagonist.

NMR spectra

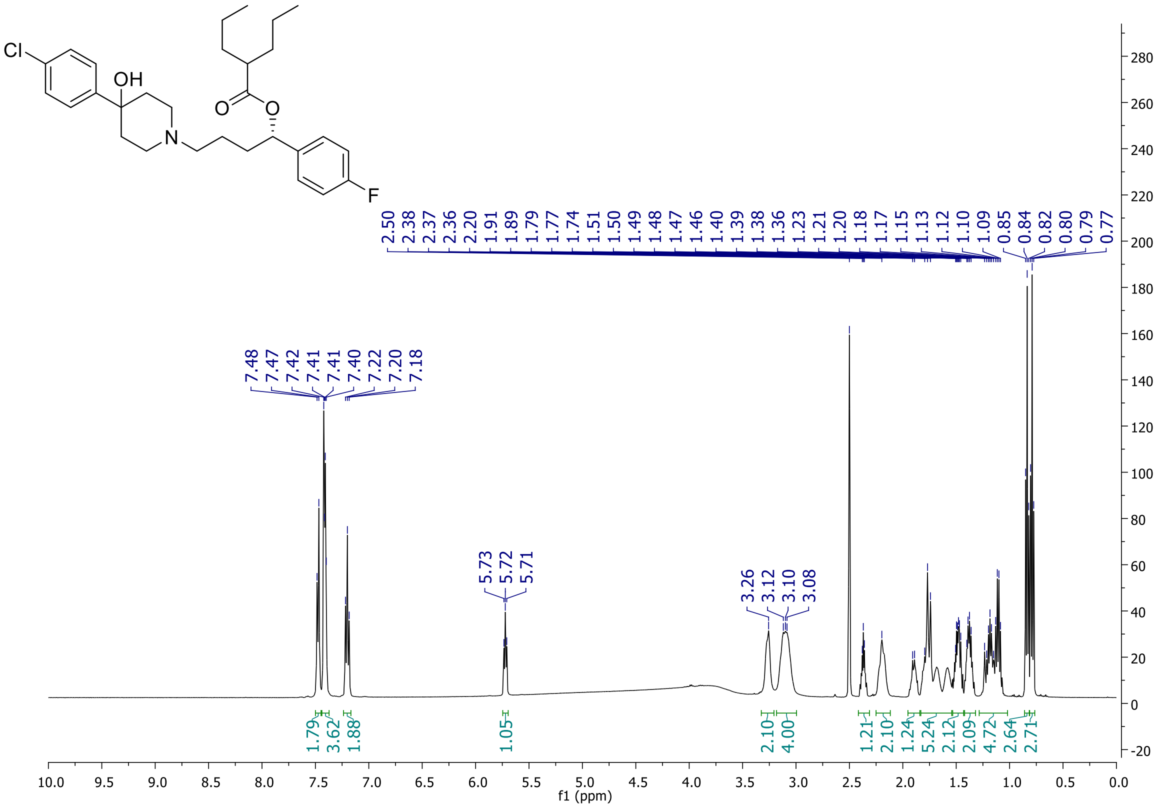

^{1}H (500 MHz, DMSO-d_6) of (*R*)-(+)-MRJF22(oxalate), [(+)-1].

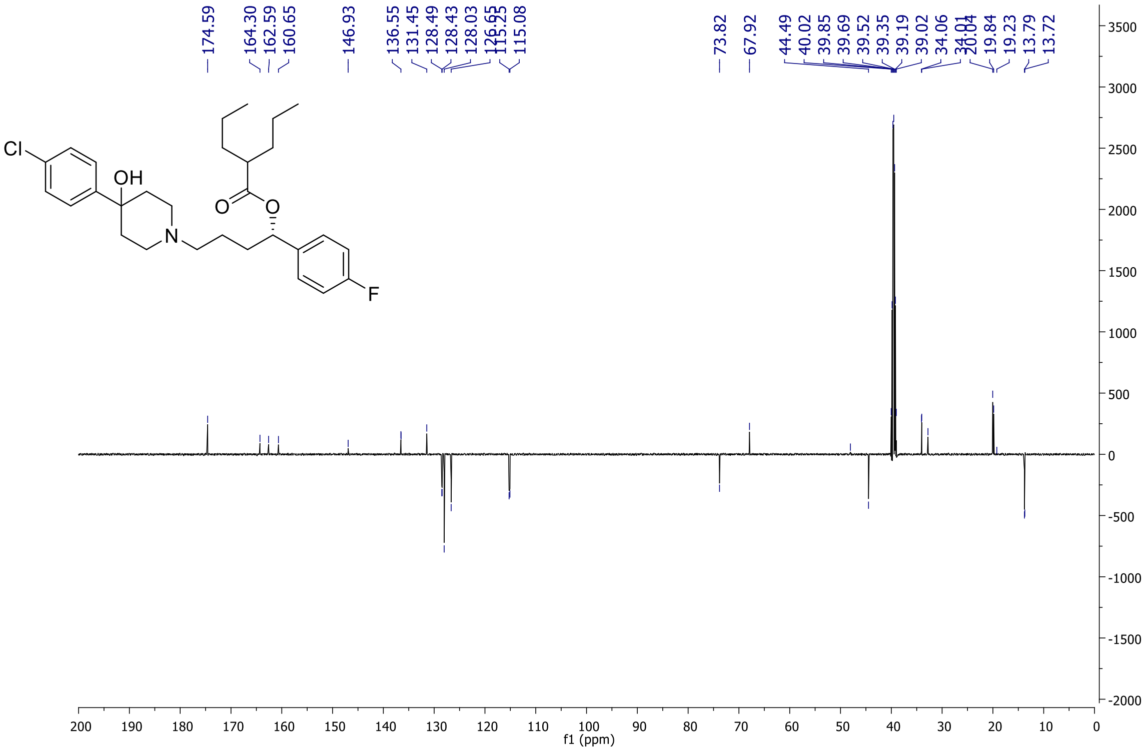

^{13}C NMR (125 MHz, DMSO-d_6) of (*R*)-(+)-MRJF22(oxalate), [(+)-1].

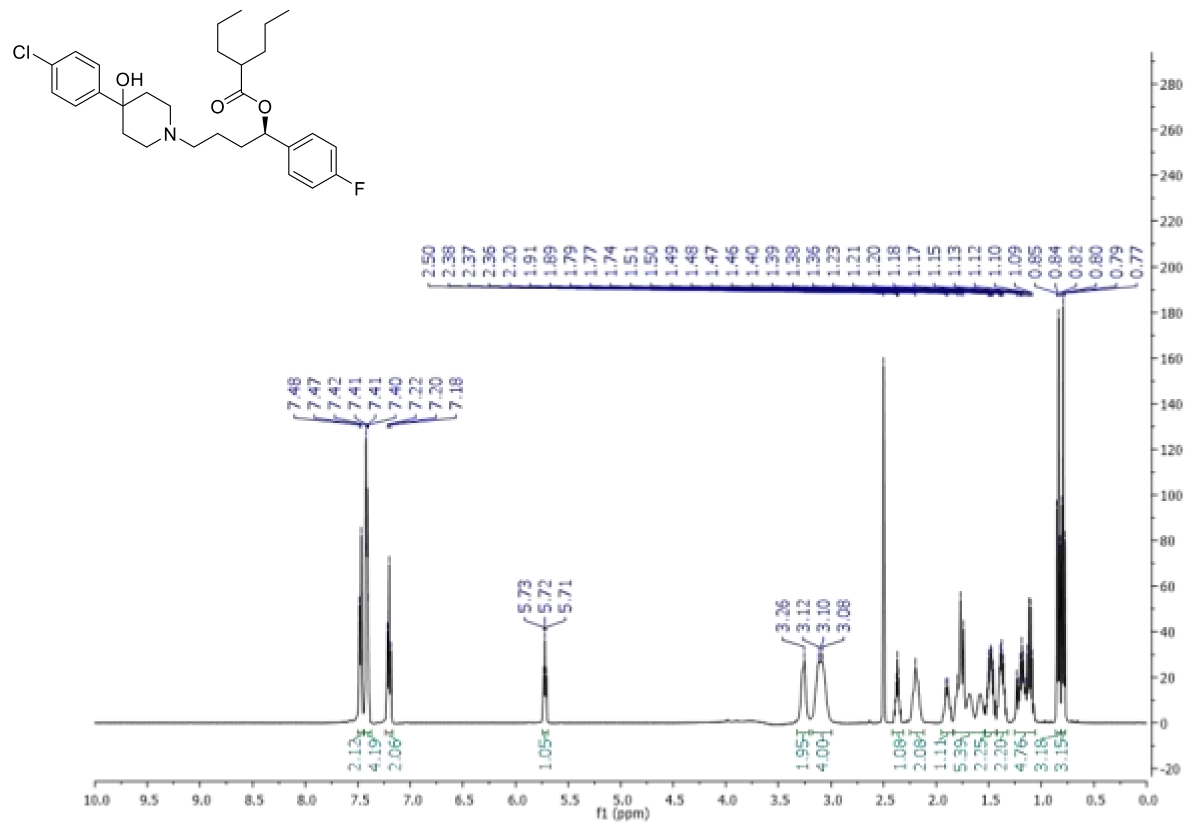

^{1}H (500 MHz, DMSO-d_6) of (*S*)-(−)-MRJF22(oxalate), [(–)-1].

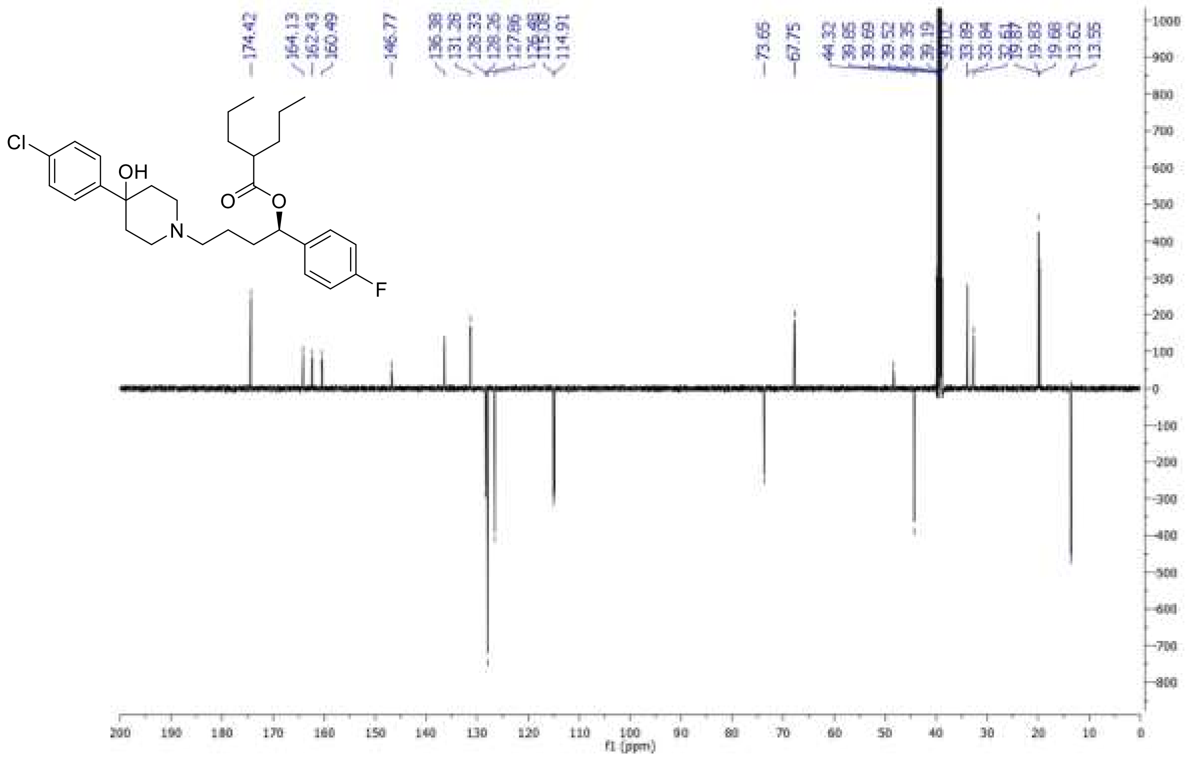

^{13}C NMR (125 MHz, DMSO-d_6) of (*S*)-(−)-MRJF22(oxalate), [(–)-1].

HR-MS spectra

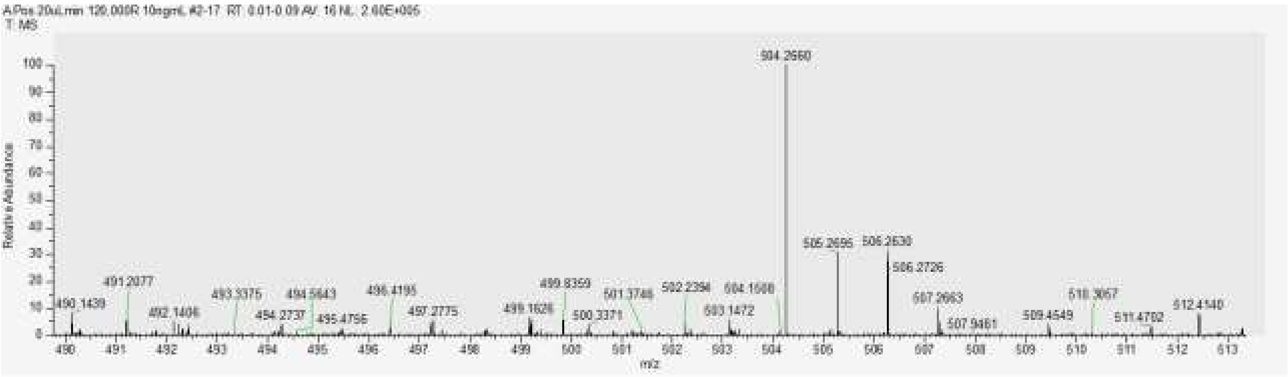

HR-MS SPECTRA of (±)-MRJF22.

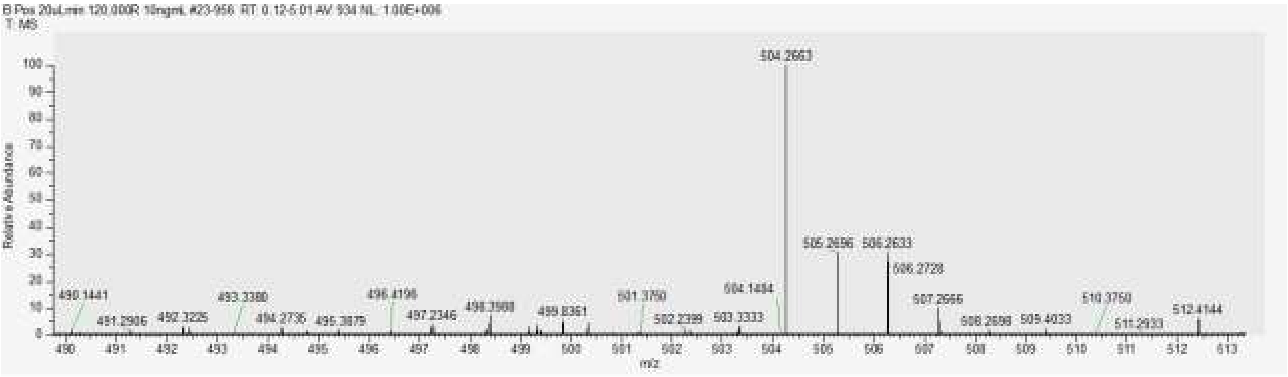

HR-MS SPECTRA of (*R*)-(+)-MRJF22 [(+)-1].

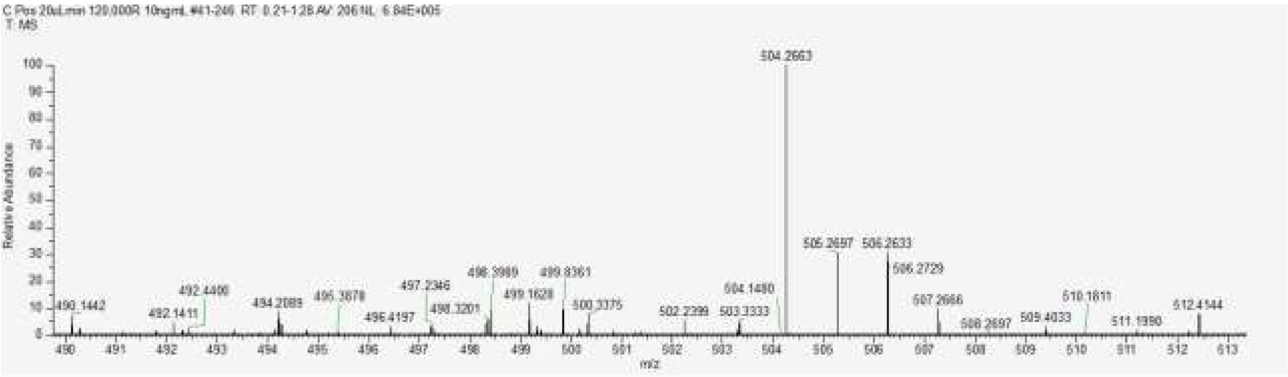

HR-MS SPECTRA of (*S*)-(+)-MRJF22 [(–)-1].
